# Metabolome and microbiome analysis revealed the effect mechanism of different feeding modes on the meat quality of Black Tibetan sheep

**DOI:** 10.3389/fmicb.2022.1076675

**Published:** 2023-01-06

**Authors:** Xue Zhang, Lijuan Han, Linsheng Gui, Sayed Haidar Abbas Raza, Shengzhen Hou, Baochun Yang, Zhiyou Wang, Ying Ma, Raafat T. M. Makhlof, Zamzam Alhuwaymil, Samah F. Ibrahim

**Affiliations:** ^1^Department of Animal Science, College of Agriculture and Animal Husbandry, Qinghai University, Xining, Qinghai, China; ^2^College of Animal Science and Technology, Northwest A&F University, Yangling, Shaanxi, China; ^3^Safety of Livestock and Poultry Products, College of Food Science, South China Agricultural University, Guangzhou, China; ^4^Department of Parasitology, Faculty of Medicine, Umm Al Qura University, Mecca, Saudi Arabia; ^5^Department of Parasitology, Faculty of Medicine, Minia University, Minya, Egypt; ^6^Organic Department, College of Science and Humanities at Al-Quway'iyah, Shaqra University, Shaqra, Saudi Arabia; ^7^Department of Clinical Sciences, College of Medicine, Princess Nourah bint Abdulrahman University, Riyadh, Saudi Arabia

**Keywords:** Black Tibetan sheep, feeding regimes, meat quality, 16S rDNA, metabolomics

## Abstract

**Introduction:**

Black Tibetan sheep is one of the primitive sheep breeds in China that is famous for its great eating quality and nutrient value but with little attention to the relationship between feeding regimes and rumen metabolome along with its impact on the muscle metabolism and meat quality.

**Methods:**

This study applies metabolomics-based analyses of muscles and 16S rDNA-based sequencing of rumen fluid to examine how feeding regimes influence the composition of rumen microbiota, muscle metabolism and ultimately the quality of meat from Black Tibetan sheep. Twenty-seven rams were randomly assigned to either indoor feeding conditions (SG, *n* = 9), pasture grazing with indoor feeding conditions (BG, *n* = 9) or pasture grazing conditions (CG, *n* = 9) for 120 days.

**Results:**

The results showed that, compared with BG and CG, SG improved the quality of Black Tibetan sheep mutton by preventing a decline in pH and increasing fat deposition to enhance the color, tenderness and water holding capacity (WHC) of the *Longissimus lumborum* (LL). Metabolomics and correlation analyses further indicated that the feeding regimes primarily altered amino acid, lipid and carbohydrate metabolism in muscles, thereby influencing the amino acid (AA) and fatty acid (FA) levels as well as the color, tenderness and WHC of the LL. Furthermore, SG increased the abundance of *Christensenellaceae R-7 group*, *[Eubacterium] coprostanoligenes group*, *Methanobrevibacter*, *Ruminococcus 2* and *Quinella*, decreased the abundance of *Lactobacillus*, *Prevotella 1* and *Rikenellaceae RC9 gut group*, and showed a tendency to decrease the abundance of *Succinivibrio* and *Selenomonas 1*. Interestingly, all of these microorganisms participated in the deposition of AAs and FAs and modified the levels of different metabolites involved in the regulation of meat quality (maltotriose, pyruvate, L-ascorbic acid, chenodeoxycholate, D-glucose 6-phosphate, glutathione, etc.).

**Discussion:**

Overall, the results suggest that feeding Black Tibetan sheep indoors with composite forage diet was beneficial to improve the mouthfeel of meat, its color and its nutritional value by altering the abundance of rumen bacteria which influenced muscle metabolism.

**Figure:**
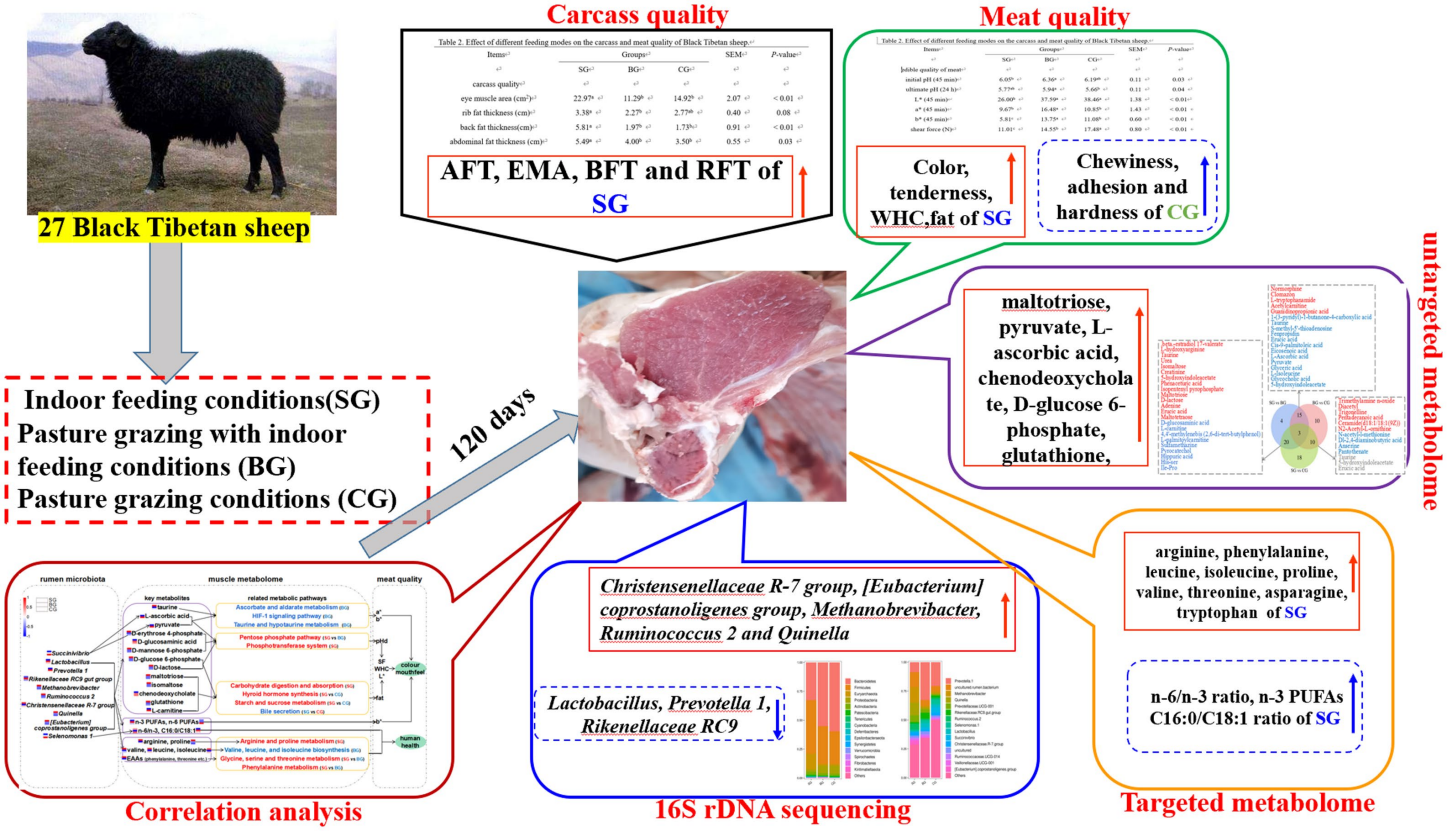
GRAPHICAL ABSTRACT

## 1. Introduction

Black Tibetan sheep, a characteristic breed of the Guinan County (Qinghai Province, China), is still raised by traditional grazing although this approach is not conducive to the protection and development of this particular livestock. In addition, overgrazing can easily degrade the grassland and lead to desertification, resulting in ecological imbalance (Peng et al., 2015). In order to protect grassland ecosystem in China as well as meet an increasing demand for high quality Black Tibetan sheep’s meat, the animals’ feeding regimes have gradually transitioned from traditional grazing to intensive feeding. This changed approach can significantly protect the ecological environment, shorten the slaughtering time and greatly increase the quantity of sheep in a short time (da Silva et al., 2020). However, such changes in feeding regimes also affect the overall health, growth characteristics and even the meat quality of sheep due to differences in diet composition (Boughalmi and Araba, 2016). For example, intensive production can increase the growth performance and carcass quality of sheep (Papi et al., 2011), while pasture grazing can improve the levels of polyunsaturated fatty acids (PUFAs) in their muscles (Wang et al., 2021). Consequently, maintaining or even improving the meat quality of Black Tibetan sheep raised in the feedlot is expected to become the core of future research.

The metabolome of muscles provides important information to characterize the nutritional and sensory properties of mutton (Muroya et al., 2020). Indeed, increasing numbers of studies have demonstrated that the levels of muscle metabolites can potentially indicate changes in meat quality. For example, a metabolite called inosine monophosphate regulates Nellore cattle beef’s tenderness, along with creatine, carnosine, and conjugated linoleic acid in the *Longissimus lumborum* (LL; de Zawadzki et al., 2017). Moreover, color stability is attributed to the presence of antioxidant or reducing compounds, such as taurine and L-glutathione (Muroya et al., 2020). Notably, in the authors’ previous study, it was found that differences between White Tibetan sheep’s meat quality under stall feeding and traditional grazing conditions were regulated by muscle differential metabolites (DMs; L-glutamate, inosine, adenosine, D-mannose, D-fructose, etc.) (Zhang et al., 2021). Therefore, these findings provide a basis for exploring the main metabolites and key metabolic pathways that regulate Black Tibetan sheep’s meat quality under different feeding regimes.

In ruminants, the rumen microbiota independently regulates host metabolism, with changes in its composition directly influencing the host’s phenotype (Zhang et al., 2022). In this context, ruminants’ feeding regime and dietary components are critical factors as they determine rumen microbiota’s composition and functions. For instance, Wang et al. (2021) discovered that artificial pasture grazing modified muscle metabolites that influence meat quality as well as muscle amino acid (AA) and fatty acid (FA) composition by modulating rumen microbiota. As such, it was a better choice for producing healthy mutton products (Wang et al., 2021). Furthermore, Du et al. (2021) found that a high-energy diet could enhance yak’s muscle quality, including fat content and shear force of the LL. This was achieved through an increase in amylolytic bacteria and their fermentation products which were required for fatty acid synthesis (Du et al., 2021). Most importantly though, the current authors previously reported that a high-energy diet could affect Black Tibetan sheep’s muscle metabolism by altering the composition of rumen microbiota, with this change improving meat quality and flavor (Zhang et al., 2022). In light of these findings, it is expected that host metabolism and animal phenotypes can be artificially modified by changing feeding regimes and diets. However, the key rumen bacteria associated with quality-related properties such as meat color and tenderness in Black Tibetan sheep are yet to be determined.

Consequently, it was hypothesized that the meat quality of this breed could be enhanced by changing feeding regimes which not only influence the rumen microbiota composition but also muscle metabolism. For this purpose, ultrahigh-performance liquid chromatography-mass spectrometry (UHPLC–MS) and gas chromatography–mass spectrometry (GC–MS) were applied to, respectively, compare changes in muscle AA and FA composition resulting from the different feeding regimes. The muscle metabolome was subsequently studied using ultrahigh-performance liquid chromatography coupled to quadrupole time-of-flight mass spectrometry (UHPLC-QTOF-MS) before determining the rumen microbiota composition based on Illumina sequencing. In addition, correlations between meat quality parameters and muscle metabolites as well as between rumen bacteria, key DMs and beneficial AAs and FAs in the LL were analyzed. This approach provided a detailed and comprehensive overview of how Black Tibetan sheep’s meat quality was influenced by different feeding regimes. The findings are expected to not only provide baseline information for enhancing Black Tibetan sheep’s meat quality by regulating rumen microbiota under an indoor feeding regime but also to provide an effective method for striking a balance between production and ecology.

## 2. Materials and methods

The study was approved by the Animal Ethics Committee of Qinghai University (QUA-2020-0709) and was carried out at the Black Tibetan Sheep Breeding Center in Guinan County of Qinghai Province, China.

### 2.1. Animal management and sample collection

Twenty-seven 120-day-old rams (Black Tibetan sheep) weighing 12.2 ± 0.92 kg were randomly assigned to one of the following feeding regimes: indoor feeding conditions (SG, *n* = 9), pasture grazing with indoor feeding conditions (BG, *n* = 9) and pasture grazing conditions (CG, *n* = 9). A composite forage with a concentrate (commercial mixed ration) to roughage (silage corn and green oat hay, 1:1) ratio of 7:3 was used as feed for the lambs from the SG group (twice a day at 08:00 and 18:00), while those in the CG and BG groups grazed (08:00–18:00) on desert grassland located in Gonghe County, Qinghai Province (36.28°N, 99.88°E). The latter contained *Achnatherum splendens* and *Agropyron splendens* (sowing proportion, 50, 50%, respectively) and included two grazing areas (687 m^2^/plot). In addition, the diet of the lambs from the BG group was supplemented with the composite forage after grazing. All the animals were subjected to a 7-day adaptation period before being fed (1.5 × 2.0 m^2^/unit, 1 lambs/unit) individually or allowed to graze (9 lambs/plot) for 120 days with *ad libitum* access to food and water. Table 1 shows the ingredients and nutritional levels of the composite forage and pasture grass.

**Table 1:** Dietary ingredients and nutritional levels of the two different feeds (dry matter basis).

Items	Composite forage	Pasture grass
Dietary ingredients (% DM)		
*Achnatherum splendens*	–	50.00
*Agropyron desertorum*	–	50.00
Corn	43.15	–
Soybean meal	2.65	–
Rapeseed meal	12.42	–
Cottonseed meal	4.58	–
Oat silage	15.00	–
Oat hay	15.00	–
Mineral salt	0.80	–
Limestone	0.80	–
Baking soda	0.10	–
Dicalcium phosphate	0.50	–
Mineral/vitamin premix^1^	5.00	–
Total	100.00	100.00
Nutritional levels (% DM)		
Digestive energy DE (MJ/kg)	12.12	–
Crude protein	13.03	5.15
Crude ash	4.62	6.82
Crude fat	5.02	3.08
Neutral detergent fiber NDF	26.72	42.28
Acid detergent fiber ADF	16.36	30.23
Calcium	0.92	0.20
Phosphorus	1.01	0.26

1 The premix provided the following per kg of diet: Fe (as ferrous sulfate) 4.5 g/kg; Cu (as copper sulfate) 1.0 g/kg; Zn (as zinc sulfate) 6.0 g/kg; Mn (as manganese sulfate 3.0 g/kg; Co (as cobalt sulfate) 0.02 g/kg, Se 0.02 g/kg; I 0.04 g/kg; VA 250000 IU/kg; VD 30000 IU/kg; VE 25000 IU/kg.

In accordance with animal welfare procedures, all animals were humanely slaughtered after fasting for 18 h on solid food and 2 h on liquid food. The left side of the carcasses, between the 12th and 13th ribs, were sampled before determining the color and pH values of each sample within 45 min. Moreover, rumen fluid was filtered through four layers of cheesecloth prior to collection into sterile containers. As required by standardized norms, slaughter, and sampling were conducted simultaneously by professionals. Following collection, samples were then immediately frozen in liquid nitrogen before being stored in the refrigerator at −80°C until needed for further analysis. A total of nine biological replicates and three technical replicates were obtained from each group to assess the concentrations of ammonia-N and volatile fatty acids (VFAs), AA and FA composition as well as carcass and meat quality. For the studies of rumen microbiota and muscle metabolome, six biological replicates were collected for each group.

### 2.2. Carcass quality analysis of Black Tibetan sheep

At the time of carcass segmentation, the carcass quality was assessed at the 12th rib. The eye muscle area (EMA) was measured with a planimeter after being drawn on sulfuric acid paper. Abdominal fat thickness (AFT), back fat thickness (BFT) and rib fat thickness (RFT) were then directly measured with vernier calipers at 127 mm, 40 mm and 110 mm from the spinal column, respectively.

### 2.3. Meat quality analysis of Black Tibetan sheep

Following a 24-h postmortem phase, standard procedures were used to assess the edible quality of meat samples. A portable pH meter, previously calibrated with standards of pH 4.0 and 6.86 and having a built-in temperature compensator, was inserted into samples to determine the initial and final pH values. A Minolta-ADCI machine was then used to determine the a* (redness), b* (yellowness) and L* (lightness) values of samples. Moreover, their shear force (SF) was assessed with a Warner-Bratzler apparatus, while the elasticity, hardness, viscosity, cohesion, adhesion and chewiness of samples were measured with a texture profile analysis (TPA) machine (CT3, Brookfield).

As previously described (Zhang et al., 2021), the samples were thawed at 4°C for 12 h to calculate the thaw loss (TL) before hanging them at the same temperature for 24 h to assess the drip loss (DL). In addition, the samples were cooked in a Thermostatic Water Bath machine at 85°C for 30 min to obtain the cooking loss (CL) and cooked meat percentage (CMP). Throughout these processes, all samples were individually packed into hermetic bags. Finally, regarding the nutritional components, standard AOAC procedures were used to determine the total ash, crude fat, crude protein and moisture content of meat samples at 48 h postmortem (Lee, 1995).

### 2.4. Untargeted metabolomics analysis of the *Longissimus lumborum*

For untargeted metabolite analysis, 50 mg of meat samples were processed as reported before (Zhang et al., 2021). This was followed by UHPLC-QTOF-MS (1,290 Infinity, Agilent) which first involved sample separation in an UHPLC. In this case, the autosampler temperature, injection volume, column temperature and flow rate were 4°C, 2 μL, 25°C and 0.5 ml/min, respectively. Furthermore, the following liquid phase gradient was applied: 0–0.5 min, the B phase was 95%; 0.5–7 min, the B phase changed linearly from 95 to 65%, then decreased linearly to 40% in 1 min before being sustained for 1 min; the B phase was then increased to 95% in 0.1 min and sustained for 2.9 min. Quality control (QC) samples were also added into the sample queues to monitor and assess the stability of the system and the reliability of the data.

MS (AB Triple TOF 6600) was subsequently used to collect the samples’ first- and second-order spectra. The chromatographic alignment, retention-time modifications and peak identification were carried out using XCMS software. Furthermore, multivariate statistical analysis was performed using SIMCA software, with heat maps subsequently generated in R. DMs, defined as those with variable importance projection (VIP) > 1 and *p* < 0.05, were also identified. Eventually, the metabolites were functionally annotated and the metabolic pathways were enriched using the Kyoto Encyclopedia of Genes and Genomes database (KEGG).^1^

### 2.5. Targeted metabolomics analysis of the *Longissimus lumborum*

#### 2.5.1. Amino acid analysis

Fifty milligrams of meat samples were processed for AA analysis as described previously (Zhang et al., 2021). For UHPLC–MS (1,290 Infinity, Agilent) detection, sample separation was performed in an UHPLC, with an autosampler temperature of 4°C, an injection volume of 1 μL, a column temperature of 40°C and a flow rate of 250 μL/min. Furthermore, the liquid phase gradient was set as follows: 0–12 min, the B phase changed linearly from 90 to 70%; 12–18 min, the B phase changed linearly from 70 to 50%; it was then decreased to 40% in 7 min and maintained for 5 min; finally, the B phase increased linearly to 90% in 0.1 min and was sustained for 6.9 min. QC samples were also added to evaluate the stability and repeatability of the system. Finally, MS (5,500 QTRAP) was set to Multiple Reaction Monitoring (MRM), while chromatographic alignment, retention-time modifications and peak identification were carried out with the MultiQuant software to determine metabolites.

#### 2.5.2. Fatty acid analysis

FA analysis was performed on 50 mg of meat samples as previous described (Zhang et al., 2021). In this case, for GC–MS-based (7,890/5975C, Agilent) detection, sample separation was carried out on a capillary column (30 m × 0.25 mm × 0.25 μM, DB-WAX) for which the programmed temperature was set to 40°C for 5 min before increasing to 220°C at a rate of 10°C/min for 5 min. Helium was used as the carrier gas in a split ratio of 10:1 and a flow rate of 1.0 ml/min, while the inlet, transfer line and ion source temperatures were 280°C, 250°C, and 230°C, respectively. QC was also performed as mentioned above. MS was set to Single Ion Monitoring (SIM) and operated in the Electron Impact Ionization Source (EIS), while electron energy was set at 70 eV. Eventually, chromatographic alignment, retention-time modifications and peak identification were carried out using MSD ChemStation software to determine metabolites.

### 2.6. Rumen function analysis

#### 2.6.1. Rumen fermentation characteristics

Following collection of the rumen fluid, a portable pH meter was used to immediately record the pH value. The concentrations of VFAs and ammonia-N were subsequently determined by GC (Shimadzu NX 2030) and the phenol-hypochlorite assay, respectively (Broderick and Kang, 1980).

#### 2.6.2. Rumen microbiota composition

Genomic DNA was extracted from rumen fluid samples using SDS technique before assessing the DNA’s purity and concentration. The V3-V4 regions of 16S rDNA genes were then amplified by PCR using specific primers under the following conditions: initial denaturation for 1 min at 98°C, followed by 30 cycles, each with denaturation for 10 s at 98°C, annealing for 30 s at 50°C and extension for 60 s at 72°C. The PCR ended with a 5-min final extension at 72°C. AxyPrep DNA Gel Extraction Kit (AXYGEN, United States) and QuantiFluor™-ST (Promega, United States) were then used to, respectively, purify and quantify the PCR products as specified by the respective manufacturers. This was followed by the preparation of a sequencing library using an Illumina Next®Ultra™DNA Library Prep Kit (NEB, United States), with both Qubit@ 2.0 Fluorometer and Agilent Bioanalyzer 2,100 systems used to assess the quality of the library. Eventually, sequencing was performed on an Illumina HiSeq2500 platform to generate 250 bp paired-end reads.

The sequencing data generated above were filtered and merged, as previously reported, to obtain effective tags (Wang et al., 2020a). UPARSE software was then used to cluster representative sequences into operational taxonomic units (OTUs) at a 97% similarity threshold before classifying the latter into various species using the RDP classifier against the SILVA database. Alpha (Chao1, Simpson, Shannon, etc.) and beta diversities (Principal Coordinate Analysis based on unweighted unifrac) were also determined in QIIME, while both Anosim and Adonis were constructed using Bray-Curtis distance matrices. Furthermore, LEfSe-based analyses allowed microbial biomarkers within each group to be quantitatively identified before using STAMP analysis to confirm the abundance of differential species between the three groups. The metabolic function of rumen microbiota was finally predicted using the KEGG database.

### 2.7. Statistical analysis

Means and standard errors of the means (SEM) were calculated with one-way analysis of variance (ANOVA) using SPSS software (16.0). Significance was set at *p* < 0.05 and tendencies were reported at 0.05 ≤ *p* ≤ 0.1. For the different feeding regimes, Pearson’s correlation coefficient was further used to determine the relationship between rumen bacteria, muscle metabolome and meat quality in Black Tibetan sheep, with significant correlations indicated by *p* < 0.05 and |r| > 0.50.

## 3. Results

### 3.1. Carcass quality

The carcass quality of Black Tibetan sheep under different feeding regimes is shown in Table 2. The AFT, EMA and BFT were higher (*p* < 0.05) in SG than in BG and CG, although these three parameters were not different between BG and CG. In addition, SG showed a tendency (*p* = 0.08) to increase the RFT. Therefore, the results indicated that Black Tibetan sheep’s carcass quality under the indoor feeding regimes was the best.

**Table 2:** Effects of different feeding modes on the carcass and meat quality of Black Tibetan sheep.

Items	Groups			SEM	*p*-value
	SG	BG	CG		
Carcass quality					
Eye muscle area (cm^2^)	22.97^a^	11.29^b^	14.92^b^	2.07	< 0.01
Rib fat thickness (cm)	3.38	2.27	2.77	0.4	0.08
Back fat thickness(cm)	5.81^a^	1.97^b^	1.73^b^	0.91	<0.01
Abdominal fat thickness (cm)	5.49^a^	4.00^b^	3.50^b^	0.55	0.03
Edible quality of meat					
Initial pH (45 min)	6.05^b^	6.36^a^	6.19^ab^	0.11	0.03
Ultimate pH (24 h)	5.77^ab^	5.94^a^	5.66^b^	0.11	0.04
L* (45 min)	26.00^b^	37.59^a^	38.46^a^	1.38	< 0.01
a* (45 min)	9.67^b^	16.48^a^	10.85^b^	1.43	< 0.01
b* (45 min)	5.81^c^	13.75^a^	11.08^b^	0.6	< 0.01
Shear force (N)	11.01^c^	14.55^b^	17.48^a^	0.8	< 0.01
Thaw loss (%)	7.24	6.44	6.25	0.58	0.21
Drip loss (%)	4.14^c^	5.10^b^	6.13^a^	0.46	< 0.01
Cooking loss (%)	17.39^c^	29.20^b^	34.69^a^	1.88	< 0.01
Cooked meat percentage (%)	76.72^a^	69.54^b^	63.40^c^	2.42	< 0.01
Hardness (g)	824.44^b^	632.56^b^	1832.67^a^	115.75	< 0.01
Elasticity (mm)	1.54^b^	2.02^a^	2.13^a^	0.11	< 0.01
Viscosity (mJ)	0.16	0.2	0.17	0.03	0.4
Adhesion (g)	252.44^b^	182.56^b^	505.78^a^	46.21	< 0.01
Cohesion	0.35	0.34	0.37	0.06	0.85
Chewiness (mJ)	6.48^b^	4.21^b^	10.02^a^	1.22	< 0.01
Nutritional quality of meat					
Moisture (%)	73.58	74.33	74.39	0.59	0.33
Ash (%)	1.10^b^	1.22^ab^	1.34^a^	0.06	< 0.01
Fat (%)	4.41^a^	3.78^ab^	3.16^b^	0.34	< 0.01
Protein (%)	21.36	21.5	21.52	0.44	0.93

^a,b,c^Different superscripts within a row indicate significantly different values for *P*-value <0.05.

### 3.2. Meat quality

The meat quality of Black Tibetan sheep under different feeding regimes is shown in Table 2. For the edible quality, BG had a higher (*p* < 0.05) initial pH value than SG as well as a higher (*p* < 0.05) ultimate pH value than CG. This showed that CG displayed the largest pH decline in the LL (within 24 h after slaughtering), followed by BG and SG, with the latter having the lowest range of pH decline. The a* and b* values were also significantly higher (*p* < 0.01) in BG than in SG and CG, whereas the L* value was significantly lower (*p* < 0.01) in SG compared with BG and CG. In addition, CG had the largest SF, DL, and CL, followed by BG, which had medium values, while SG had the lowest ones (*p* < 0.01). Conversely, SG had a higher CMP than CG, while BG was in the median range (*p* < 0.01). In addition, the chewiness, adhesion, and hardness were significantly lower (*p* < 0.01) in SG and BG in comparison with CG, whereas the elasticity was considerably lower (*p* < 0.01) in SG. Finally, as far as the nutritional components of the LL were concerned, the fat content was considerably higher (*p* < 0.01) but the ash content was considerably lower (*p* < 0.01) in SG than in CG. As a result, SG showed better meat edible quality [e.g., color, tenderness, and water holding capacity (WHC)] as well as greater meat fat deposition.

### 3.3. Analysis of muscle untargeted metabolome

**Figure 1:**
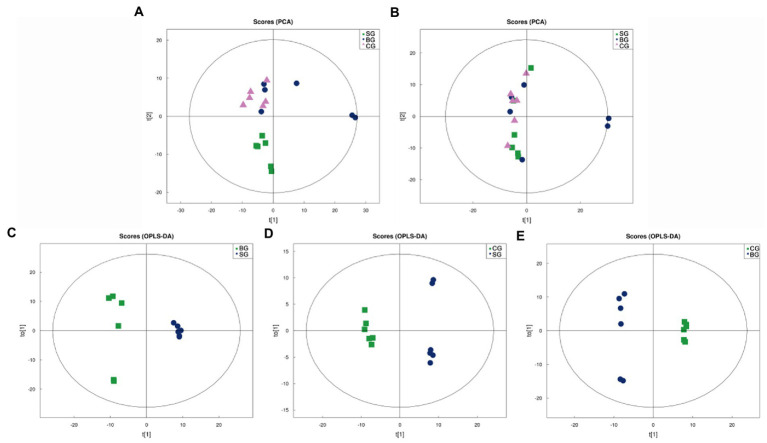
PCA score plots of all samples in the ESI+ **(A)** and ESI− **(B)** modes as well as OPLS-DA score plots of the comparison between SG and BG **(C)**, between SG and CG **(D)**, and between BG and CG **(E)** in the ESI+ mode. t[1] represents the principal component 1, t[2] **(A,B)** and to[1] **(C–E)** represent the principal component 2, and the ellipse represents the 95% confidence interval.

As shown in Figures 1A,B, the Principal Component Analysis (PCA) scores for the three groups were plotted under positive (ESI+) and negative (ESI−) ion modes, respectively. In this case, the R^2^X of the ESI+ and ESI− modes for the three groups were 0.609 and 0.532, respectively, and hence, an ESI+ mode was selected for subsequent analysis as this was more useful and reliable. It was noted that, under this mode, the SG samples clustered separately from those of BG and CG, although no clear separation was observed for the latter two. Therefore, to further optimize the three groups’ separation and focus on the metabolic variations between the LL, Orthogonal Partial Least Squares-Discriminant Analysis (OPLS-DA) was used to build more intensive and accurate models for the analysis of the three sets of meat samples, with the resulting OPLS-DA score plots shown in Figures 1C–E (ESI+). In this case, OPLS-DA revealed good within-group aggregation along with between-group separation. R^2^X, R^2^Y, and Q^2^ of the OPLS-DA models are also summarized in Supplementary Table S1, and all of the models showed acceptable goodness-of-fit and goodness-of-prediction. Therefore, the results indicated that the metabolic pattern in Black Tibetan sheep’s LL was significantly altered by different feeding regimes.

**Figure 2:**
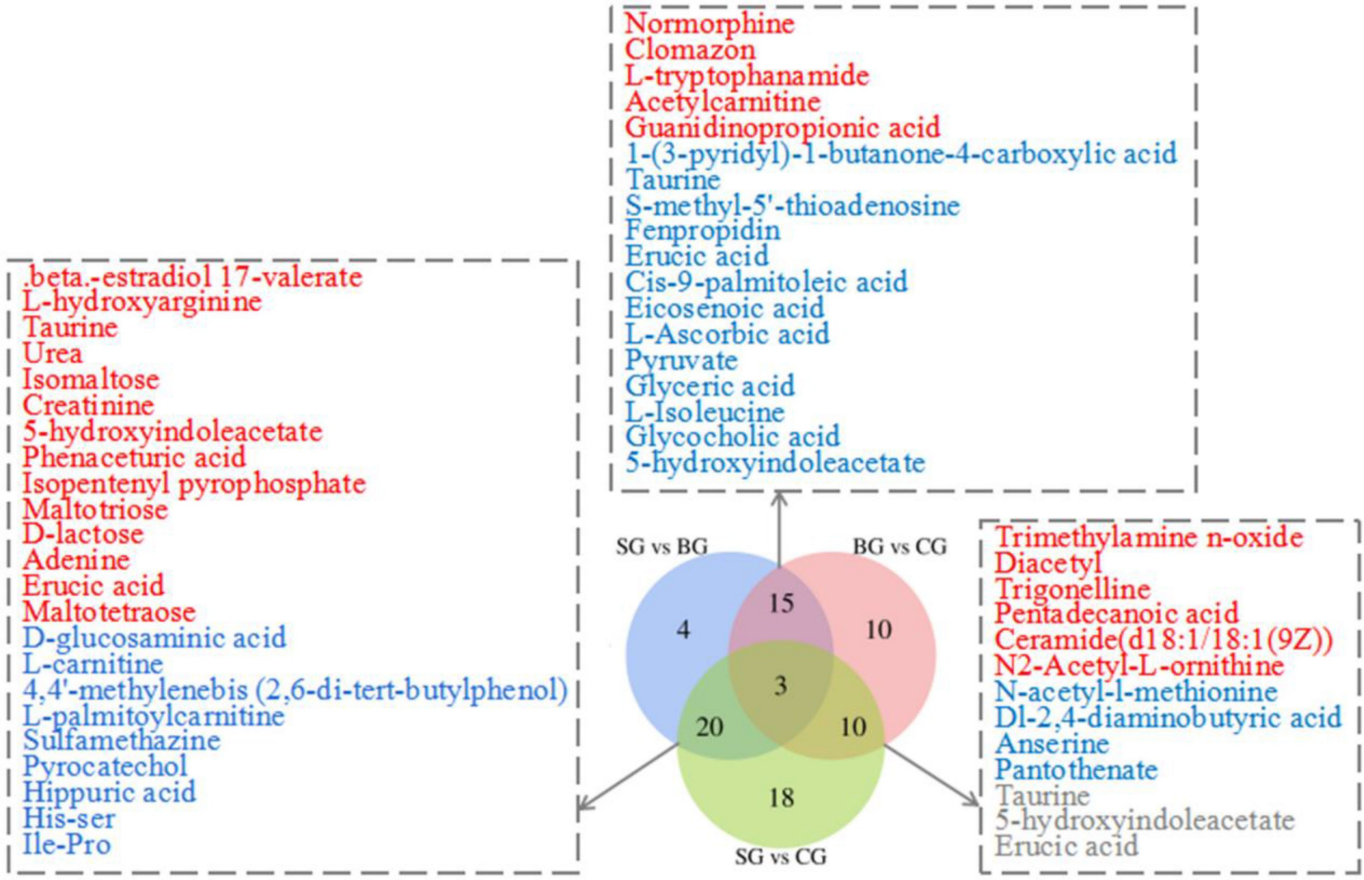
Venn diagram illustrates the overlap of DMs connected with KEGG metabolic pathways for three sets of comparisons (SG vs. BG; SG vs. CG; BG vs. CG) in the *Longissimus lumborum*. The color red and blue represent the upregulation and downregulation of metabolites, respectively.

By applying the threshold (VIP > 1, *p* < 0.05) for DMs’ identification under both the ESI+ and ESI− modes, a total of 171 DMs were identified across the three groups, of which 80 were connected with KEGG metabolic pathways (Figure 2). More specifically, pairwise comparisons revealed 42 DMs (18 and 24 in the positive and negative modes, respectively) between SG and BG, 51 DMs (25 and 26 in the positive and negative modes, respectively) between SG and CG and 38 DMs (20 and 18 in the positive and negative modes, respectively) between BG and CG. Compared with BG and CG, 23 metabolite levels were also significantly different in SG (14 DMs were significantly higher, and 9 DMs were lower). Furthermore, 18 metabolite levels were different in BG (13 DMs were significantly lower, whereas 5 DMs were higher) compared with the other groups, while for the CG group, the levels of 10 metabolites were significantly different (4 DMs were significantly lower, and 6 DMs were higher). Overall, most of these DMs were lipids, AAs, carbohydrates and their derivatives.

**Figure 3:**
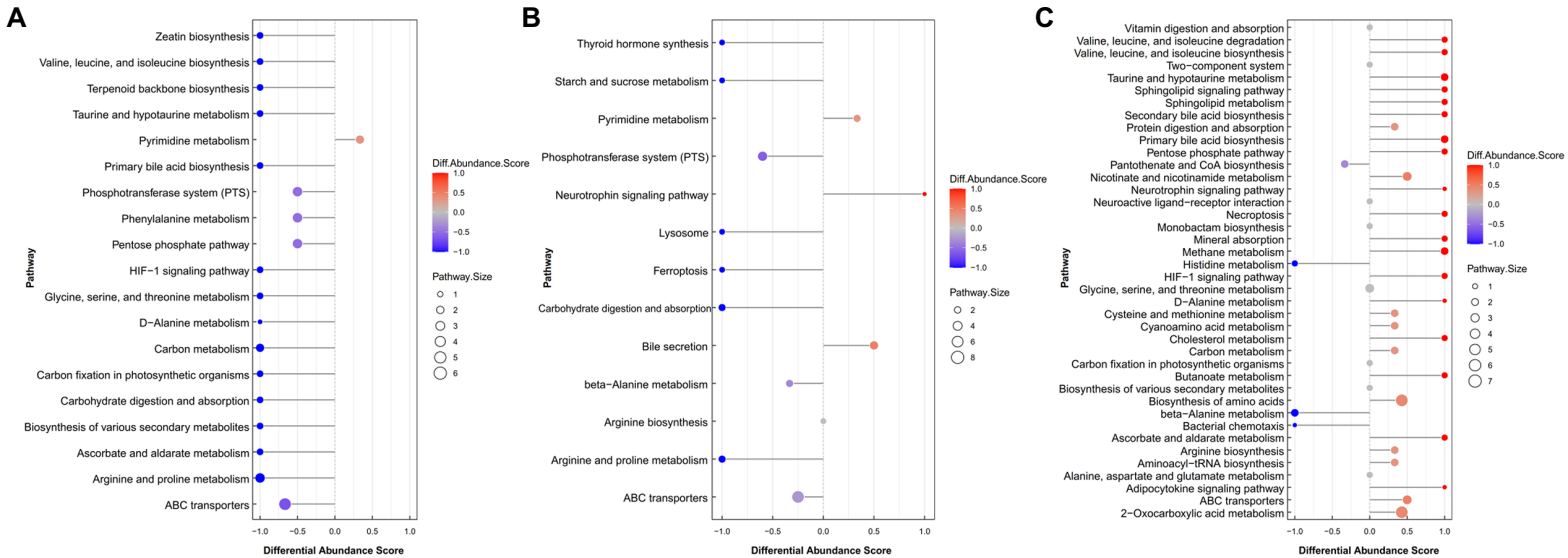
The DA score maps of all differential metabolic pathways for the comparison between SG and BG **(A)**, between SG and CG **(B)** and between BG and CG **(C)** in the *Longissimus lumborum*. The DA score captures the average and gross changes for all metabolites in a pathway. Scores of 1 and − 1, respectively, indicate a decrease and an increase in all measured metabolites in the pathway for SG **(A,B)** and BG **(C)**.

To further determine the differential metabolic pathways in Black Tibetan sheep’s LL after different feeding regimes, the Differential Abundance Score (DA score) of each comparable group was analyzed. As shown in Figure 3A, compared with BG, 18 metabolic pathways were upregulated in SG (DA score > 0.5, *p* < 0.05), with the key ones being phosphotransferase system (PTS), pentose phosphate pathway, ascorbate and aldarate metabolism, carbohydrate digestion and absorption, HIF-1 signaling pathway and six amino acid metabolisms (taurine, hypotaurine metabolism, etc.). Among the metabolites involved in these key pathways, the levels of 3-hydroxyphenylacetic acid, D-erythrose 4-phosphate, phenaceturic acid, urea, taurine, L-isoleucine, maltotriose, L-hydroxyarginine, L-ascorbic acid, glyceric acid, D-lactose, pyruvate and creatinine were all higher in SG, whereas those of D-glucosaminic acid and hippuric acid were lower (Supplementary Table S3). On the other hand, compared with CG, only two metabolic pathways were downregulated in SG, while seven were upregulated (DA score > 0.5, *p* < 0.05). Bile secretion was the main downregulated pathway in SG, unlike starch and sucrose metabolism, arginine and proline metabolism, carbohydrate digestion and absorption, thyroid hormone synthesis and PTS which were all upregulated (Figure 3B). Among the metabolites involved in these key pathways, the levels of D-glucose 6-phosphate, maltotriose, L-hydroxyarginine, isomaltose, creatinine, D-mannose 6-phosphate, glutathione, D-lactose and urea were all higher in SG, while those of D-glucosaminic acid, chenodeoxycholate, L-carnitine and choline were lower (Supplementary Table S3). Finally, comparison between CG and BG showed that 3 metabolic pathways were downregulated and 20 were upregulated in the former (DA score > 0.5, *p* < 0.05). In this case, the key upregulated pathways included the HIF-1 signaling pathway, pentose phosphate pathway, ascorbate and aldarate metabolism, four amino acid metabolisms (taurine, hypotaurine metabolism, etc.) and four lipid metabolisms (sphingolipid metabolism, etc.). On the other hand, beta-alanine metabolism and histidine metabolism were downregulated in CG (Figure 3C). Among the metabolites involved in these key pathways, the levels of methylmalonic acid, glyceric acid, L-isoleucine, sphingomyelin (d18:1/18:0), taurine, ceramide [d18:1/18:1(9Z)], glycocholic acid, pyruvate, taurocholate and L-ascorbic acid were all higher in CG, while those of anserine, pantothenate and aspartic acid were lower (Supplementary Table S3).

### 3.4. Analysis of muscle targeted metabolome

#### 3.4.1. Amino acid composition

As shown in Table 3, the concentrations of total amino acids (TAAs) and non-essential amino acids (NEAAs) in the LL did not differ between the three groups, although SG showed a tendency to increase the levels of essential amino acids (EAAs; *p* = 0.09), arginine (*p* = 0.07) and threonine (*p* = 0.08). Similarly, the levels of tyrosine, asparagine, taurine and aminoadipic acid were higher (*p* < 0.05) in SG than in BG. In addition, the levels of proline and valine were higher (*p* < 0.05) in SG than in BG and CG, the concentrations of methionine, phenylalanine, leucine, isoleucine and hydroxyproline were higher (*p* < 0.01) in SG and CG than in BG, and BG had a higher (*p* < 0.05) concentration of tryptophan compared with SG and CG.

**Table 3:** Effects of different feeding modes on the AA composition in the *Longissimus lumborum* of Black Tibetan sheep (μg/g tissue).

Items	Groups	SEM	*P*-value		
	SG	BG	CG		
Arginine	108.46	66.26	90.77	14.61	0.07
Methionine	5.87^a^	4.07^b^	5.71^a^	0.35	<0.01
Phenylalanine	44.25^a^	26.90^b^	39.47^a^	3.22	<0.01
Tyrosine	47.02^a^	27.64^b^	38.63^ab^	5.86	0.04
Leucine	74.33^a^	47.42^b^	66.43^a^	5.74	<0.01
Isoleucine	23.84^a^	17.04^b^	22.02^a^	1.32	<0.01
Proline	77.08^a^	40.81^b^	35.98^b^	10.40	0.01
Valine	54.07^a^	37.86^b^	43.56^b^	4.26	0.02
Threonine	57.63	22.92	45.43	12.53	0.08
Asparagine	43.93^a^	20.48^b^	34.95^ab^	6.26	0.03
Tryptophan	39.51^b^	54.88^a^	42.05^b^	4.91	0.04
Hydroxyproline	36.43^a^	10.80^b^	36.59^a^	7.94	0.03
Taurine	1014.74^a^	365.16^b^	678.34^ab^	188.75	0.04
Choline	22.14	190.63	50.89	64.24	0.08
Aminoadipic acid	26.24^a^	9.19^b^	16.09^ab^	5.31	0.05
EAAs^1^	344.21	255.26	300.03	33.19	0.09
NEAAs	4781.98	3533.80	4892.30	798.59	0.25
TAAs	5126.19	3789.06	5192.33	830.41	0.24

^a,b^Different superscripts within a row indicate significantly different values for *P*-value <0.05. Complete amino acid profile is provided in Supplementary Table S4. EAAs, essential amino acids; NEAAs, non-essential amino acids; TAAs, total amino acids.

1 EAAs = (leucine + methionine + valine + isoleucine + threonine + phenylalanine + lysine + tryptophan).

#### 3.4.2. Fatty acid composition

As noted from Table 4, the levels of total fatty acids (TFAs), saturated fatty acids (SFAs), monounsaturated fatty acids (MUFAs) and PUFAs, including the ratio of PUFAs/SFAs (P/S) in the LL, were not different between the three groups even though the ratios of n-6/n-3 and C16:0/C18:1 were significantly affected by different feeding regimes. In particular, BG showed a tendency (*p* = 0.05) to increase the concentration of n-3 PUFAs as well as CG showed a tendency (*p* = 0.07) to increase the concentration of n-6 PUFAs. The C16:0/C18:1 ratio was higher (*p* < 0.05) in BG than in SG, while the n-6/n-3 ratio was higher (*p* < 0.01) in SG and CG than in BG. In addition, SG had a higher (*p* < 0.01) C20:1 N9 concentration than BG and CG. Similarly, BG had higher (*p* < 0.05) C20:5 N3 and C22:5 N3 concentrations than SG as well as a higher (*p* < 0.05) C22:6 N3 concentration than both SG and CG. Conversely, CG had a higher (*p* < 0.05) concentration of C18:3 N6 compared with BG.

**Table 4:** Effects of different feeding modes on the FA composition in the *Longissimus lumborum* of Black Tibetan sheep (μg/g tissue).

Items	Groups			SEM	*P*-value
	SG	BG	CG		
C18:2 N6	520.10	515.18	706.96	76.09	0.08
C18:3 N6	6.20^a^	3.19^b^	7.01^a^	1.09	0.03
C18:3 N3	26.54	30.90	52.72	9.59	0.07
C20:1 N9	22.48^a^	10.94^b^	13.22^b^	2.09	<0.01
C20:3 N6	18.68	28.17	25.19	3.65	0.10
C20:4 N6	2.81	2.07	2.56	0.24	0.06
C20:3 N3	187.83	310.29	190.08	47.26	0.07
C20:5 N3	14.40^b^	44.02^a^	21.83^ab^	9.45	0.05
C23:0	0.88	0.72	1.17	0.17	0.10
C22:5 N3	39.09^b^	88.59^a^	69.11^ab^	12.97	0.02
C22:6 N3	6.77^b^	22.67^a^	8.90^b^	4.48	0.02
TFAs	6707.74	5737.86	6832.17	2164.09	0.86
SFAs	3010.23	2747.13	3434.54	1250.24	0.86
MUFAs	2839.16	1921.01	2279.78	931.81	0.63
PUFAs	858.35	1069.72	1117.85	137.12	0.21
n-3 PUFAs	274.63	496.47	342.64	71.72	0.05
n-6 PUFAs	583.73	573.25	775.21	78.04	0.07
PUFAs/SFAs	0.34	0.45	0.39	0.16	0.80
n-6 PUFAs/n-3 PUFAs	2.12^a^	1.19^b^	2.26^a^	0.17	<0.01
C16:0/C18:1	0.58^b^	0.87^a^	0.77^ab^	0.09	0.05

^a,b^Different superscripts within a row indicate significantly different values for *p*-values <0.05. Complete fatty acid profile is provided in Supplementary Table S5. TFAs, total fatty acids, SFAs, saturated fatty acids; MUFAs, monounsaturated fatty acids; PUFAs, polyunsaturated fatty acids; n-3 PUFAs, omega-3 polyunsaturated fatty acids; n-6 PUFAs, omega-6 polyunsaturated fatty acids.

### 3.5. Rumen function

#### 3.5.1. Analysis of rumen fermentation characteristics

Fermentation characteristics in Black Tibetan sheep’s rumen under the three different feeding regimes were as shown in Table 5, with no differences in the pH value observed for the three groups. However, SG had a higher (*p* < 0.01) ammonia-N concentration than BG and CG. For VFAs, SG had higher (*p* < 0.01) butyrate and valerate levels than BG and CG. Besides, SG had a lower (*p* < 0.01) ratio of acetate to propionate (A/P) than BG and CG.

**Table 5:** Effects of different feeding modes on rumen fermentation characteristics of Black Tibetan sheep.

Items	Groups			SEM	*P*-value
	SG	BG	CG		
pH	5.71	5.59	5.75	0.12	0.38
Ammonia-N (mmol/L)	21.68^a^	8.84^b^	8.42^b^	2.40	<0.01
Acetate (mmol/L)	60.10	65.91	66.96	5.02	0.35
Propionate (mmol/L)	23.42	20.27	21.53	1.30	0.07
Butyrate (mmol/L)	18.37^a^	11.10^b^	12.57^b^	1.22	<0.01
Valerate (mmol/L)	1.35^a^	0.69^c^	0.95^b^	0.11	<0.01
Total VFAs (mmol/L)	107.71	97.95	102.52	7.27	0.42
A/P	2.57^b^	3.22^a^	3.14^a^	0.16	<0.01

^a,b,c^Different superscripts within a row indicate significantly different values for *P*-value <0.05. VFAs, volatile fatty acids; A, acetate; P, propionate.

#### 3.5.2. Analysis of rumen microbiota composition

**Figure 4:**
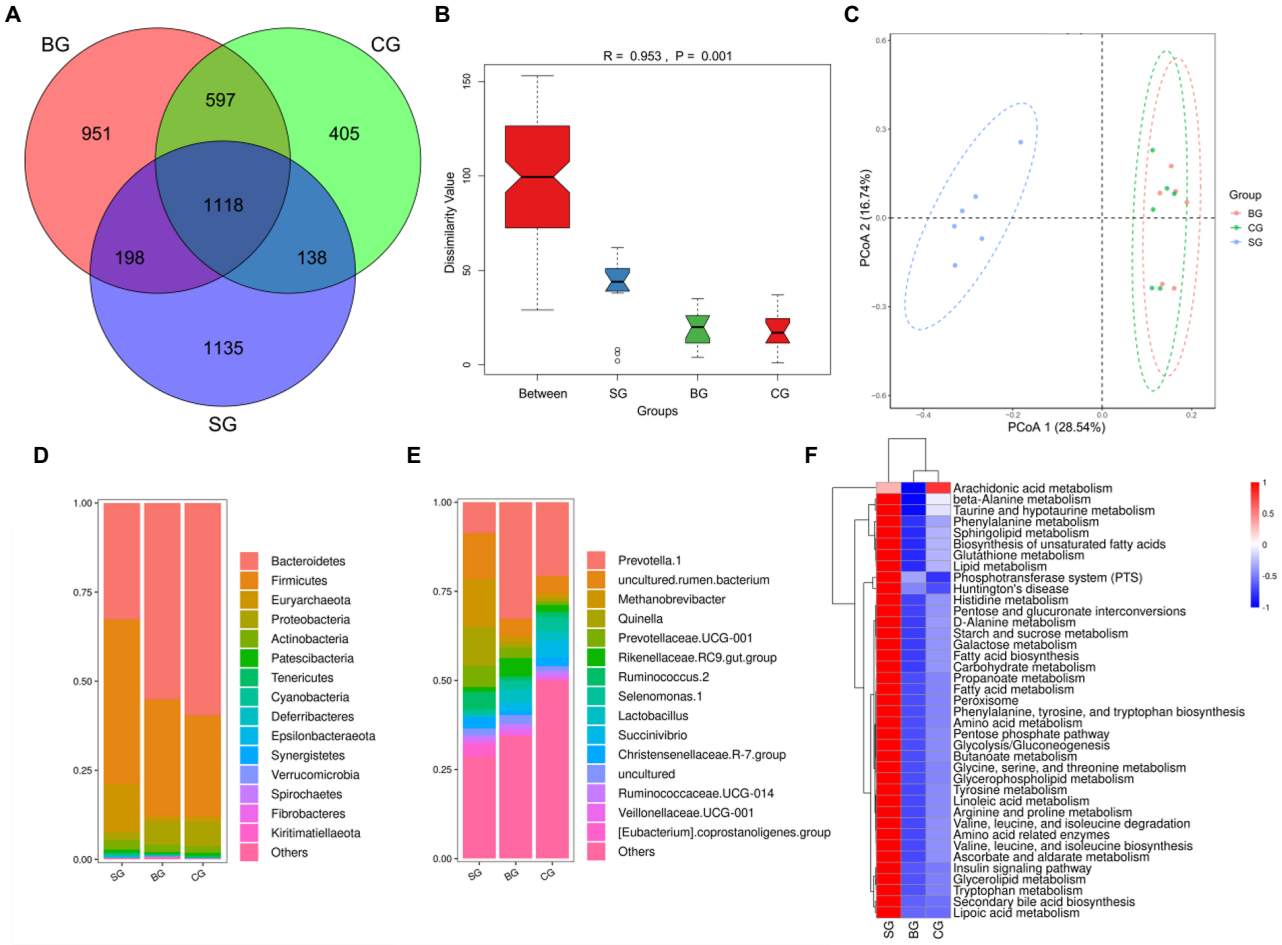
Venn diagram of OTUs showing the overlap of rumen microbiota between the three groups **(A)**. Anosim analysis **(B)** and PCoA plot **(C)** of the samples’ rumen microbiota. The relative abundance of the 15 most abundant taxa at the phylum **(D)** and genus **(E)** levels among the three groups (as a percentage of the total sequences). Heatmap predicting the important differences in rumen functions **(F)**.

As shown in Figure 4A, 1118 OTUs were obtained across the three groups, with 1,135, 951 and 405 being assigned only to SG, BG, and CG, respectively. At the same time, significant differences were noted in bacterial diversity and richness between the three groups of samples (Supplementary Table S2). More specifically, BG had higher (*p* < 0.01) Shannon and Simpson values than SG and CG, while CG had lower (*p* < 0.01) Chao1 and Ace than SG and BG. Anosim analysis (Figure 4B) and PCoA plot (Figure 4C) further revealed significant differences and good separation between SG and the other two groups, although similar results were not obtained when comparing BG and CG. Overall, these findings highlight the changes in Black Tibetan sheep’s rumen bacteria in response to differences between the three feeding regimes. Furthermore, 372 bacterial genera from 22 phyla were identified, with Figures 4D,E showing the top 15 most abundant genera and phyla, respectively.

The main differences in phyla and genera of rumen bacteria between the three groups are shown in Table 6. At the phylum level, compared with BG and CG, SG had more Euryarchaeota, Deferribacteres, Epsilonbacteraeota, Firmicutes and Synergistetes (*p* < 0.01) but less Bacteroidetes (*p* < 0.01). Moreover, Cyanobacteria, Spirochaetes and Kiritimatiellaeota were more abundant (*p* < 0.01) in BG than in SG and CG. At the genus level, *Quinella*, *Methanobrevibacter*, *Ruminococcus 2* and *[Eubacterium] coprostanoligenes group* were more abundant (*p* < 0.01) in SG compared with BG and CG, while *Lactobacillus* was less abundant (*p* < 0.01). At the same time, compared with SG and CG, BG had more *Rikenellaceae RC9 gut group* and *Prevotella 1* (*p* < 0.01) but less *Christensenellaceae R-7 group* (*p* < 0.05). Finally, CG had a higher abundance of *Prevotella 1* (*p* < 0.01) compared with SG, while the latter showed a tendency to decrease the abundance of *Selenomonas 1* (*p* = 0.10) and *Succinivibrio* (*p* = 0.06). Based on the results, functional prediction of Black Tibetan sheep’s rumen microbiota suggested that composite forage diet promoted the metabolism of carbohydrates, AAs, lipids and other substances in the rumen (Figure 4F).

**Table 6:** The main differential rumen bacteria at the phylum and genus levels among the three groups (accounting for the relative abundance in top 10).

Items	Groups			SEM	*P*-value
	SG	BG	CG		
Phylum level (%)					
Bacteroidetes	32.66^b^	55.10^a^	59.60^a^	3.96	<0.01
Firmicutes	46.32^a^	32.72^b^	28.25^b^	4.48	<0.01
Euryarchaeota	13.44^a^	1.57^b^	1.59^b^	3.46	<0.01
Cyanobacteria	0.03^b^	0.26^a^	0.09^b^	0.05	<0.01
Deferribacteres	0.32^a^	0.00^b^	0.00^b^	0.09	<0.01
Epsilonbacteraeota	0.24^a^	0.01^b^	0.00^b^	0.09	0.03
Synergistetes	0.15^a^	0.06^b^	0.03^b^	0.02	<0.01
Spirochaetes	0.03^b^	0.15^a^	0.04^b^	0.03	<0.01
Fibrobacteres	0.00	0.17	0.02	0.07	0.05
Kiritimatiellaeota	0.02^b^	0.10^a^	0.03^b^	0.01	<0.01
Genus level (%)					
*Prevotella 1*	8.64^c^	32.72^a^	20.63^b^	3.10	<0.01
*Methanobrevibacter*	13.40^a^	1.56^b^	1.57^b^	3.46	<0.01
*Quinella*	10.98^a^	1.61^b^	0.73^b^	0.64	<0.01
*Rikenellaceae RC9 gut group*	1.23^b^	5.16^a^	1.90^b^	0.92	<0.01
*Ruminococcus 2*	5.08^a^	1.24^b^	1.63^b^	1.37	0.03
*Selenomonas 1*	1.30	2.43	3.91	1.13	0.10
*Lactobacillus*	0.83^b^	3.69^a^	2.68^a^	0.78	<0.01
*Succinivibrio*	0.11	2.31	4.61	1.73	0.06
*Christensenellaceae R-7 group*	3.06^a^	1.16^b^	2.48^a^	0.58	0.02
*[Eubacterium] coprostanoligenes group*	3.30^a^	0.31^b^	0.19^b^	0.53	<0.01

^a,b,c^Different superscripts within a row indicate significantly different values for *P*-value <0.05.

### 3.6. Correlation analysis

#### 3.6.1. Correlation between muscle untargeted metabolome and meat quality parameters

**Figure 5:**
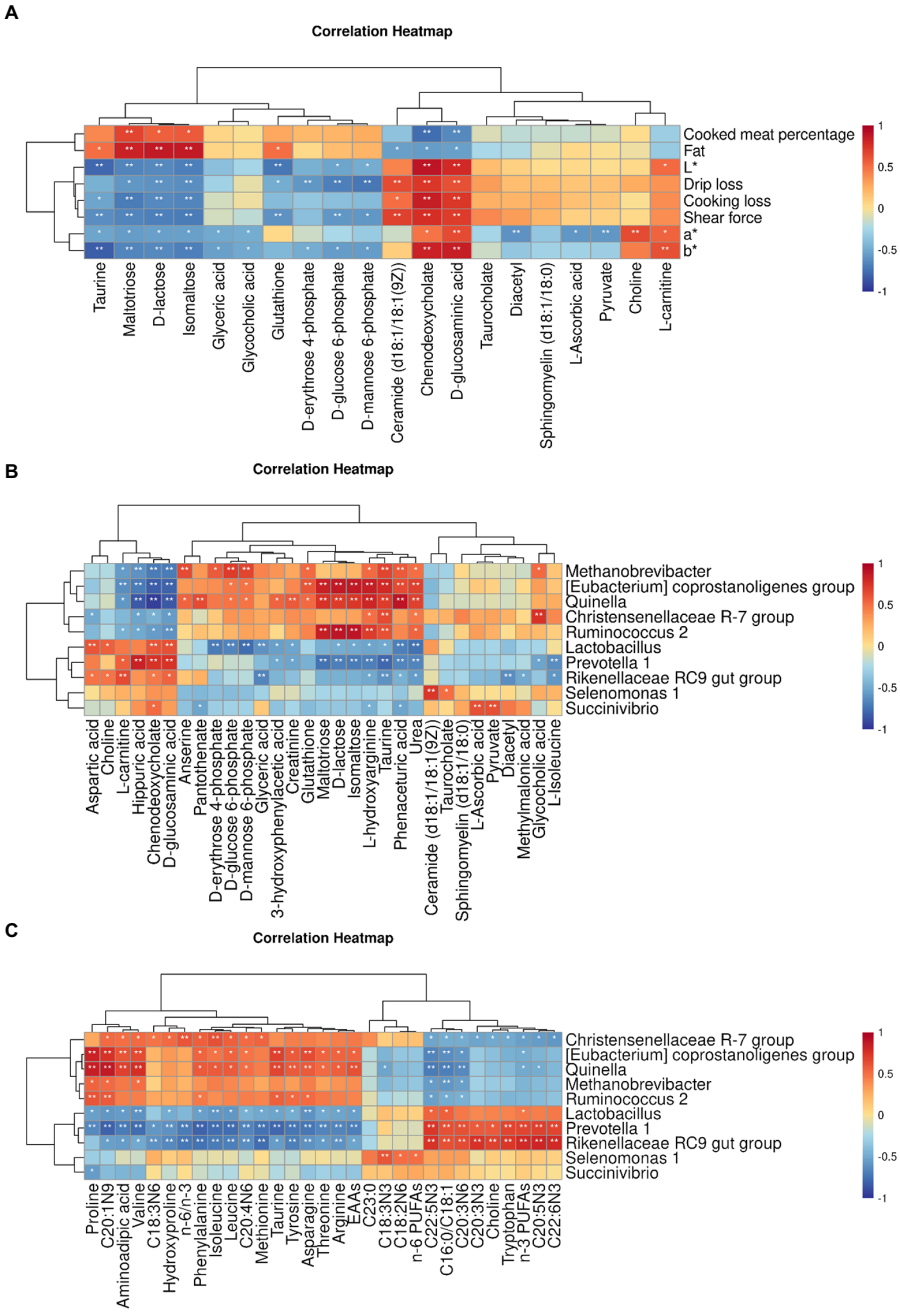
Pearson’s correlation heatmap between muscle untargeted metabolome and meat quality parameters **(A)**, between rumen bacteria and muscle DMs **(B)** and between rumen bacteria and the AA and FA profiles **(C)**. The color red and blue represent positive and negative correlations, respectively. The color intensity is proportional to the correlation values. *p-value <0.05 and **p-value <0.01.

The correlation between meat quality and muscle metabolism was assessed using the data from untargeted metabolome and the phenotypic parameters of Black Tibetan sheep’s LL under different feeding regimes (Figure 5A). In this context, L*, a*, b*, SF, DL, and CL were positively correlated with D-glucosaminic acid and chenodeoxycholate but negatively correlated with maltotriose, D-lactose and isomaltose. Conversely, CMP and fat were negatively correlated with D-glucosaminic acid and chenodeoxycholate but positively correlated with maltotriose, D-lactose and isomaltose. In addition, L* was negatively correlated with D-glucose 6-phosphate and glutathione but positively correlated with L-carnitine. Negative correlations were also noted for a* with pyruvate, taurine and L-ascorbic acid as well as for DL with D-erythrose 4-phosphate, D-glucose 6-phosphate and D-mannose 6-phosphate. In the case of glutathione, it was positively correlated with fat content, while negative correlations were observed for glutathione, D-glucose 6-phosphate and D-mannose 6-phosphate with SF as well as between taurine and b*. Overall, bile secretion, pentose phosphate pathway, PTS, carbohydrate digestion and absorption as well as sucrose and starch metabolism significantly affected the values of L*, a*, b*, SF, DL, CL, CMP, and fat content in the LL. Taurine and hypotaurine metabolism, ascorbate and aldarate metabolism and HIF-1 signaling pathway also significantly affected the a* and b* values of the LL, with thyroid hormone synthesis also affecting SF, L* value and fat content.

#### 3.6.2. Correlation of rumen bacteria with muscle amino acids, fatty acids, and differential metabolites

A strong connection was found between rumen bacteria and muscle DMs (Figure 5B) as well as the profiles of AAs and FAs (Figure 5C) in the LL. In particular, in the case of *Prevotella 1*, *Rikenellaceae RC9 gut group* and *Lactobacillus*, a positive correlation was noted with D-glucosaminic acid, chenodeoxycholate and L-carnitine, while a negative correlation was observed for *[Eubacterium] coprostanoligenes group*, *Methanobrevibacter*, *Quinella*, *Ruminococcus 2* and *Christensenellaceae R-7 group* with those metabolites. A positive correlation was also noted for *[Eubacterium] coprostanoligenes group*, *Methanobrevibacter* and *Quinella* with glutathione as well as for *Succinivibrio* with pyruvate and L-ascorbic acid. In addition, *Lactobacillus* was negatively correlated with D-erythrose 4-phosphate, D-glucose 6-phosphate and D-mannose 6-phosphate, whereas *Methanobrevibacter* was positively correlated with those metabolites. In the case of *Quinella*, *Ruminococcus 2* and *[Eubacterium] coprostanoligenes group*, a positive correlation was observed with maltotriose, D-lactose, isomaltose and taurine, while *Prevotella 1* was negatively correlated with those metabolites. Regarding the AA profiles, *Rikenellaceae RC9 gut group*, *Prevotella 1* and *Lactobacillus* were negatively correlated with arginine, phenylalanine, leucine, isoleucine, proline, valine, threonine, asparagine, tryptophan, taurine and the total EAA content, whereas *Quinella* and *[Eubacterium] coprostanoligenes group* were positively linked to those metabolites. Furthermore, *Christensenellaceae R-7 group* was positively correlated with leucine, isoleucine and valine. Finally, as far as FA profiles were concerned, positive correlations were observed between *Selenomonas 1* and n-6 PUFAs as well as between *Christensenellaceae R-7 group* and the n-6/n-3 ratio. In addition, *Rikenellaceae RC9 gut group* and *Prevotella 1* were negatively linked to the n-6/n-3 ratio but, along with *Lactobacillus*, they were positively correlated with n-3 PUFAs and the C16:0/C18:1 ratio. On the other hand, *[Eubacterium] coprostanoligenes group*, *Quinella* and *Christensenellaceae R-7 group* were negatively linked to n-3 PUFAs, with these organisms, along with *Methanobrevibacter* and *Ruminococcus 2*, being also negatively correlated with the C16:0/C18:1 ratio.

## 4. Discussion

Feeding regimes have a significant effect on sheep’s carcass quality (Prache et al., 2021), and, as reported in previous findings (da Silva et al., 2020; Morales Gómez et al., 2021), this study also found that Black Tibetan sheep raised indoors with composite forage diet had the best carcass quality. This had been attributed to a lower A/P ratio in the rumen alongside a combination of higher energy intake and lower energy expenditure (Boughalmi and Araba, 2016). Indoor feeding regimes can provide a higher dietary nutritional level and digestive energy for Black Tibetan sheep as, under this type of feeding, the animals require less exercise, have lower energy consumption and gain weight at a faster rate, resulting in better production performance (Aurousseau et al., 2004). In contrast, those under pasture grazing consume a lot of energy due higher physical activity, resulting in less fat deposition in muscles and subsequently, poorer carcass quality (Turner et al., 2014).

Meat color, as an important indicator of meat quality, can affect consumers’ purchasing behavior and the shelf life of fresh meat (Hussain et al., 2021). In this context, higher a* as well as lower L* and b* values are indicative of better muscle color within a certain range. It has been reported that the L* value was positively correlated with muscle WHC, and a change in the refractive index of muscle surface as a result of water exudation was the main reason for the increase in the L* value (Kim et al., 2011). Therefore, for this study, the better WHC justified the lower L* value in SG compared with BG and CG. At the same time, muscle WHC is affected by the rate of pH decline and the content of intermuscular fat. Indeed, the slower the pH decline rate and the higher the intermuscular fat content, the better the muscle WHC (Moreno et al., 2020), and interestingly, similar observations were made in this study. Moreover, BG had higher a* and b* values than SG and CG, and this could have been linked to the degree of muscle oxidation. In fact, myoglobin combines with oxygen to form bright red oxymyoglobin, resulting in a higher a* value, while for b*, a change in value is associated with lipid oxidation (Hernández Salueña et al., 2019). Therefore, indoor feeding regimes can improve muscle WHC by slowing down the rate of pH decline and increasing the fat deposition, thereby reducing the L* value. On the other hand, pasture grazing with indoor feeding regimes might reduce the antioxidant capacity of muscles, resulting in increased a* and b* values.

Mutton products’ tenderness determines their acceptability and satisfaction among consumers (O'Quinn et al., 2018). In this context, SF represents an indicator that directly reflects a change in tenderness (Bendall, 1978). Furthermore, according to some studies, feedlot cattle tend to be more tender than pasture cattle (Wicks et al., 2019; Torrecilhas et al., 2021). Similarly, a previous study has shown that the muscle SF of White Tibetan sheep which had been raised indoors with composite forage diet was lower compared with those which had been raised through a traditional grazing regime (Zhang et al., 2021). Despite the above factors, meat tenderness is actually dependent on a number of other factors, including the collagen and fat content of muscular tissues, the length of sarcomeres and the rate of pH decline (Veiseth et al., 2004). A rapid decrease in pH causes muscle contraction and thus, reduces muscle tenderness (Choi et al., 2005). However, breaking muscle fibers is easier to implement during the chewing process, especially with an increase in intramuscular fat content which is translated into a decrease in muscle SF (Chambaz et al., 2003). Therefore, the lower SF in SG compared with BG and CG in this study might be attributed to the slower rate of pH decline and a higher fat deposition in the LL.

Glycolysis is predominant in post-slaughtered muscle due to the cessation of blood circulation and the interruption of oxygen supply (Hou et al., 2020). The accumulation of lactic acid from this process is the main reason of the pH decline in muscles (Choe et al., 2008). Through the phosphorylation and transfer of carbon source, PTS reduces glucose phosphorylation, resulting in a reduction in cAMP (cyclic adenosine monophosphate) concentrations. As a result, this decreases glycogen metabolism by inhibiting the APK (cAMP-dependent protein kinase) signaling pathway and inactive glycogen phosphorylase’s phosphorylation (Kim et al., 2004). In this study, compared with BG and CG, PTS was significantly upregulated in SG, hence suggesting that glycogen metabolism was inhibited in this group. Additionally, hexokinase (HK), which converts glucose into D-glucose 6-phosphate, is the first rate-limiting enzyme in the glycolytic pathway as the accumulation of D-glucose 6-phosphate decreases the activity of HK (Hingst et al., 2019). Furthermore, part of the glucose in the postmortem muscle is involved in the pentose phosphate pathway, with glucose-6-phosphate dehydrogenase (G6PD) being the key enzyme that transfers glucose metabolism to this pathway (Tang, 2019). In the present study, SG had a higher D-glucose 6-phosphate level compared with CG as well as an upregulated pentose phosphate pathway compared with BG. It was, therefore, speculated that SG could have inhibited glycolysis by decreasing HK’s activity and increasing G6PD’s activity in the LL to slow down the rate of pH decline.

Interestingly, the L* value, SF and WHC indexes (DL, CL, and CMP) were significantly linked with D-lactose and D-glucosaminic acid in this study, while SF and DL were negatively correlated with D-mannose 6-phosphate and D-glucose 6-phosphate. A negative correlation was also noted between DL and D-erythrose 4-phosphate as well as between the L* value and D-glucose 6-phosphate. All these metabolites were involved in PTS (SG vs. the other two groups) and the pentose phosphate pathway (SG vs. BG). Overall, it is suggested that indoor feeding regimes could have regulated the rate of pH decline in the LL by inhibiting glycolysis, thereby improving the muscle color, SF and WHC of Black Tibetan sheep. In particular, these events were achieved by upregulating PTS (SG vs. the other two groups), the pentose phosphate pathway (SG vs. BG) and the D-glucose 6-phosphate deposition level (SG vs. CG) in SG. However, further research (e.g., glycogen and glucose content, glycolytic potential, and enzyme activity in the LL) would still be required to support the above theory.

According to previous studies, an increase in carbohydrate metabolism can provide the host with more substrate and energy for fat synthesis in muscles (Smith et al., 2018; Du et al., 2021). Conversely, bile promotes the digestion and absorption of lipids by the liver and regulates cholesterol metabolism, thereby reducing fat deposition in muscle tissues (Boyer, 2013). In this context, thyroid hormones play an important role as they are involved in multiple metabolic pathways, including lipid catabolism, lipid anabolism and body weight regulation in mammalian systems (Sinha et al., 2019). Consequently, attenuated thyroid hormone signaling results in decreased lipid utilization by the liver, which in turn increases fat deposition in muscle tissues (Ferrandino et al., 2017). This study found that the muscle metabolites involved in carbohydrate metabolism (maltotriose, D-lactose, and isomaltose), bile secretion (chenodeoxycholate and glutathione) and thyroid hormone synthesis (glutathione) were strongly correlated with fat content. As such, the upregulation of carbohydrate digestion and absorption (SG vs. the other two groups), starch and sucrose metabolism (SG vs. CG) and thyroid hormone synthesis (SG vs. CG), along with the downregulation of bile secretion (SG vs. CG), were potential indicators of fat deposition. At the same time, the L* value, SF and WHC indexes (DL, CL and CMP) were significantly associated with maltotriose, D-lactose, isomaltose and chenodeoxycholate in the current study. In particular, the L* value and SF were negatively correlated with D-glucose 6-phosphate and glutathione. Similarly, DL was negatively correlated with D-glucose 6-phosphate, while the L* value was positively correlated with L-carnitine. All of these metabolites are involved in metabolic pathways connected with fat deposition. Overall, it is suggested that indoor feeding regimes could have regulated the fat deposition process in the LL by upregulating carbohydrate digestion and absorption (SG vs. the other two groups), starch and sucrose metabolism (SG vs. CG) and thyroid hormone synthesis (SG vs. CG), while downregulating bile secretion (SG vs. CG) to improve muscle color, SF and WHC of Black Tibetan sheep.

The oxidative stability of muscle tissues mainly depends on the balance between antioxidants and oxidative mediators (Nair et al., 2014), with the former consisting of non-enzymatic antioxidants as well as antioxidant enzymes (Wood and Enser, 1997). Vitamin C (VC), also known as ascorbic acid, is a non-enzymatic antioxidant in animals. VC can effectively scavenge reactive oxygen species (ROS) and alleviate the inhibitory effects of stress response on the activities of antioxidant enzymes, such as catalase (CAT) and glutathione peroxidase (GSH-Px), to enhance animals’ antioxidant and anti-stress capacity (Shojadoost et al., 2021). Taurine, as a sulfur-containing amino acid, can improve the antioxidant and anti-stress capacity of animals by regulating the enzymatic antioxidant defense system in cells (e.g., increasing the activities of CAT and GSH-Px in response to stress; Baliou et al., 2021). Besides, taurine exerts antioxidant effects by activating various signaling pathways (e.g., *Nrf2*) in cells (Bai et al., 2016). It is worth noting that hypoxic environments can also induce the expression of hypoxia-inducible factor (HIF-1α) in humans and animals. HIF-1α regulates the activities of antioxidant enzymes [GSH-Px, heme oxygenase (HO^−1^), etc.], thereby increasing the antioxidant capacity and reducing the oxidative stress damage of the host (Yeligar et al., 2010). Moreover, high expression of HIF-1α might also increase the expression of most antioxidant proteins in muscle tissues by enhancing the transcription and protein expression of the *Nrf2* gene, thereby improving the organisms’ antioxidant capacity (Yeligar et al., 2010). In this study, the downregulation of ascorbate and aldarate metabolism, taurine and hypotaurine metabolism as well as the HIF-1 signaling pathway suggested a decrease in the levels of ascorbate, taurine and HIF-1α in BG compared with SG and CG. At the same time, the a* value was negatively correlated with pyruvate, L-ascorbic acid and taurine, with a similar negative correlation noted between the b* value and taurine. All these metabolites were likely to be involved in ascorbate and aldarate metabolism, taurine and hypotaurine metabolism and HIF-1 signaling pathway. Overall, it is suggested that pasture grazing with indoor feeding regimes could have reduced the antioxidant capacity of the LL by downregulating the above metabolic processes and signaling pathway, thereby negatively impacting muscle color. However, more evidence (e.g., antioxidant capacity, lipid oxidation degree, and hemoglobin content in the LL) would be required to ascertain this theory.

Amino acids are important ingredients that add flavor and nutritional value to mutton (Cai et al., 2010). For example, branched-chain AAs (leucine, isoleucine, and valine) promote muscle growth by stimulating mRNA translation *via* mTORC1 (mammalian target of rapamycin complex 1) signaling (Rohini et al., 2018). Similarly, eucine, isoleucine and valine regulate carbohydrate and lipid metabolism, inhibit proteolysis and increase protein synthesis (Rohini et al., 2018), while arginine produces enzymes and proteins that oxidize substrates to regulate muscle oxidation (Wu, 2009). Furthermore, as a substrate, proline is used in the synthesis of pyruvate and glucose, which can subsequently be used to characterize muscle collagen content, while threonine gives mutton a sweet taste, making it more likeable to consumers (Madruga et al., 2010). Among their other functions, AAs further contribute to the meat’s aroma by interacting with carbonyl compounds during cooking (Dinh et al., 2018). In this study, SG promoted the deposition of proline and arginine in the LL by upregulating the metabolism of these AAs compared with BG and CG. At the same time, BG reduced the deposition of branched-chain AAs in the LL by downregulating valine, leucine and isoleucine biosynthesis compared with SG and CG. In fact, there was a tendency for higher concentration of threonine in the LL of the SG group through the upregulation of glycine, serine and threonine metabolism. It was previously reported that stall feeding regimes could increase EAA deposition in sheep’s LL (Zhang et al., 2021), and interestingly, the similar result was observed in this study. Thus, an indoor feeding regime is more likely to enhance Black Tibetan sheep’s meat flavor and promote human health.

The composition and content of FAs are important indicators of the nutritional value of meat products. In addition, the UFAs present in mutton, especially n-3 PUFAs and n-6 PUFAs, are beneficial to human health (Simopoulos, 2008). Natural pasture grass can inhibit biohydrogenation in rumen and increase the PUFA levels in muscle tissues (Ponnampalam et al., 2016). This could actually justify a tendency for higher concentrations of n-3 PUFAs in BG and n-6 PUFAs in CG as observed in this study. However, meat with high PUFA level is also more prone to lipid oxidation during storage and processing, resulting in rancidity, odor and the deterioration of meat color. Consequently, the meat’s edible quality and nutritional value are reduced, with their shelf life also shortened and altogether, these factors affect the market acceptance of meat (Scollan et al., 2014). For instance, Priolo et al. (2001) discovered that low oxidative stability of meat resulted in its pronounced browning. In the current study, increasing PUFA level in BG (n-3 PUFAs) and CG (n-6 PUFAs) might have impaired the oxidative stability of muscles and reduce meat quality. This could actually justify the lower b* value and better meat color observed for SG. An important determinant of meat’s nutritional value is its n–6/n–3 ratio, which assesses whether the meat is healthy for consumers (Simopoulos, 2002). In this context, mutton’s n–6/n–3 value needs to be approximately four to maintain good cardiovascular health in human (Molendi-Coste et al., 2011). Moreover, the risk of diabetes in humans is directly proportional to the dietary C16:0/C18:1 ratio. Since decreasing the C16:0/C18:1 value improves insulin sensitivity in humans (Saglimbene et al., 2017), this could be an effective method to reduce the blood sugar concentration and control diabetes. Thus, although a tendency for higher concentrations of n–3 PUFAs in BG and n–6 PUFAs in CG was observed in this study, these characteristics actually increased the risk of lipid oxidation. Meanwhile, SG had a higher n–6/n–3 ratio closer to the recommended value as well as a lower C16:0/C18:1 ratio compared with BG. This confirmed the higher meat quality obtained from indoor feeding regimes.

Increasing numbers of studies have reported that changes in rumen microbiota associated with muscle metabolites can be used to explore the mechanism for improving the meat quality of ruminants under different feeding regimes (Zhang et al., 2022). In this study, all rumen samples were dominated by Firmicutes and Bacteroidetes as reported in previous findings (Cunha et al., 2011; Li et al., 2012). Firmicutes can promote fiber breakdown (Evans et al., 2011), while Bacteroidetes can help digest complex carbohydrates (Spence et al., 2006). Furthermore, differences in rumen bacteria at the genus level between the three groups and their association with metabolite deposition in the LL were detected. In this study, *Prevotella*, the dominant genus in all samples, comprised 8.64–32.72% of all rumen bacteria and this was consistent with previous reports (Patel et al., 2014; Xue et al., 2017). *Prevotella* was reported to play a significant role in the efficient utilization of hemicelluloses, starch degradation, and protein and peptide metabolism (Wallace et al., 1997). For instance, in a study conducted by Zhou et al., *Rikenellaceae RC9 gut group* fermented carbohydrates or proteins, potentially improving the metabolism of lipids (Liyuan et al., 2018). Similarly, another study revealed that *Succinivibrio*, an amylolytic bacteria, fermented succinate into propionate to supply energy for the host (Zhang et al., 2018). In addition, *Selenomonas* is capable of fermenting glucose to produce acetate and propionate, with *Christensenellaceae R-7 group* also involved in acetate production. *Ruminococcus* belongs to the Firmicutes phylum and mainly degrades fibrous substances, while *Lactobacillus* have been used as probiotics (Xu et al., 2017). Unexpectedly, it was found that Black Tibetan sheep’s rumen from the SG group had a higher abundance of Firmicutes but a lower abundance of Bacteroidetes compared with BG and CG. In particular, indoor feeding regimes decreased the abundance of *Prevotella 1* (vs. the other two groups), *Lactobacillus* (vs. the other two groups) and *Rikenellaceae RC9 gut group* (vs. BG), showed a tendency for lower abundance of *Selenomonas 1* and *Succinivibri*, while increasing that of *Methanobrevibacter*, *Ruminococcus 2*, *Quinella* and *[Eubacterium] coprostanoligenes group* compared with BG and CG as well as that of *Christensenellaceae R-7 group* compared with BG. However, these results were inconsistent with previous findings (Wang et al., 2020a,b), and this could have been due to the differences between the ingredients of composite feedstuff used in this study and those used in other works.

Rumen bacteria may indirectly affect the muscle’s metabolite deposition by interacting with the host (Wang et al., 2021). It was found that some rumen bacteria were significantly correlated with the deposition of AAs and FAs as well as lipid metabolites such as L-carnitine, acetylcarnitine, linoleic acid, and linolenic acid in muscles (Wang et al., 2021). For instance, Huws et al. (2011) discovered that *Prevotella* was related to rumen lipid metabolism, while Wang et al. (2020a) discovered that *Rikenellaceae RC9 gut group* could modulate the metabolism of lipids, energy and glucose in the host because a high abundance of *Rikenellaceae RC9 gut group* in grazing sheep’s rumen could promote the deposition of glycodeoxycholic acid, alpha-linolenic acid and glycocholic acid in mutton. Interestingly, the results of the current study were consistent with the above reports. Besides *Selenomonas 1*, all differential rumen bacterial genus were significantly correlated with muscle metabolites involved in the regulation of meat quality including maltotriose, D-lactose, pyruvate, D-erythrose 4-phosphate, L-ascorbic acid, D-glucosaminic acid, isomaltose, chenodeoxycholate, D-mannose 6-phosphate, taurine, D-glucose 6-phosphate, L-carnitine and glutathione. Moreover, *Selenomonas 1*, *[Eubacterium] coprostanoligenes group*, *Prevotella 1*, *Quinella*, *Rikenellaceae RC9 gut group*, *Lactobacillus* and *Christensenellaceae R-7 group* were significantly correlated with muscle FA and AA composition. More importantly though, predicting the rumen functions of Black Tibetan sheep under the three feeding regimes suggested that indoor feeding regimes promoted the metabolism of carbohydrates, AAs, lipids and other substances in the rumen. This was, in fact, consistent with the enrichment of metabolic pathways based on muscle DMs.

**Figure 6:**
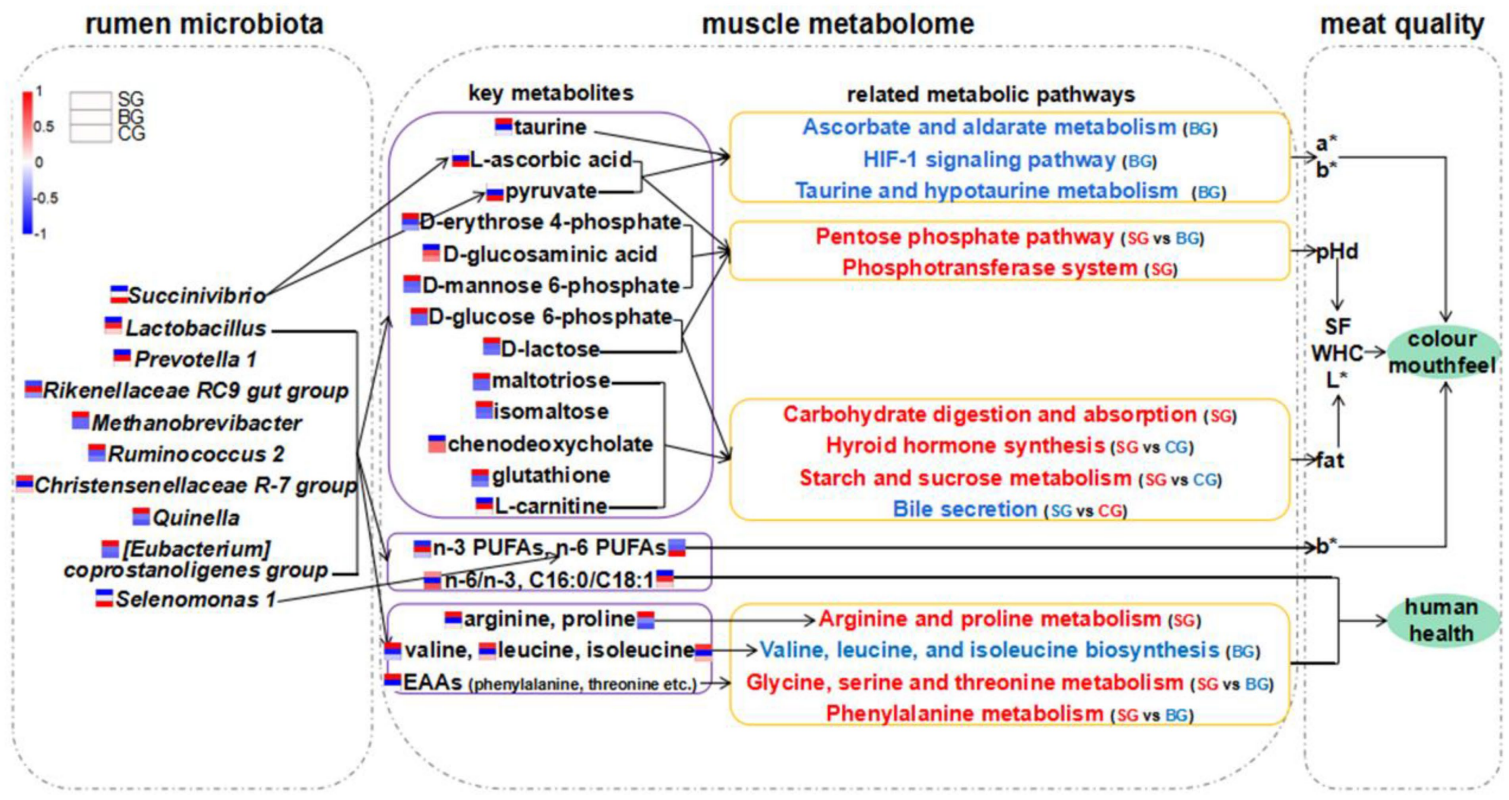
Hypothesized scheme pathways and potential mechanisms related to the changes of rumen microbiota, muscle metabolome and meat quality. Blue and red colors indicate significantly downregulated and upregulated metabolic pathways in each comparison, respectively. Expression levels of AAs, FAs, metabolites and bacteria in SG, BG, and CG were presented based on heatmaps. Red represents high expression and blue represents low expression. Complete heatmap analysis for these expression levels is provided in Supplementary Figure S1. pHd, the extent of pH decline (within 24 h after slaughter); SF, shear force; WHC, water holding capability.

SCFAs are reportedly the end-products of fermentation performed by rumen microbiota (Saleem et al., 2013). At the same time, the rumen microbiome mainly provides substrates and energy, in the form of VFAs, to the host for muscle metabolism (Nathani et al., 2015). Hence, changes in dietary nutrient components not only modify the available fermentation substrates but also rumen SCFA profiles, which subsequently affect the metabolic pathways of rumen microbiota and the LL (Ghimire et al., 2017). For example, *Ruminiclostridium_6* and *U29-B03* might participate in carbohydrate metabolism to produce VFAs, thereby facilitating IMF deposition to promote tenderness in muscles (Du et al., 2021). In this study, changes in the rumen bacteria composition of Black Tibetan sheep could explain the higher concentrations of butyrate and valerate in the SG group, with these changes resulting in the upregulated metabolism of carbohydrates, AAs, lipids and other substances in the rumen and LL. Thus, it was proposed that composite forage diet could regulate metabolite deposition in the LL by altering the rumen microbiota composition as well as the SCFA profiles to improve meat quality (Figure 6). This is because a change in feeding regime induces a change in the amount of exercise, feed intake and dietary nutrient components, with these being the main factors that affect rumen microbial fermentation and muscle metabolite deposition (Chikwanha et al., 2018). In the present study, the tendency for higher concentrations of EAAs in the LL of the SG group were likely due to the high concentrations of EAAs in the composite forage, along with the higher protein utilization efficiency of rumen microbiota (Matthews et al., 2019). In addition, it was very likely that the tendency for higher concentrations of n-3 PUFAs and n-6 PUFAs in the BG and CG groups were due to the high concentrations of PUFAs in pasture grass as well as the low biohydrogenation of the ingested PUFAs (Fernandez-Turren et al., 2020). Finally, the significant differences in rumen microbiota composition between the three groups also reflected a change in feeding pattern or diet (Shabat et al., 2016). However, more studies would be required to reveal the connection between diet components and the changes in rumen microbiota, muscle metabolome and rumen metabolites.

## 5. Conclusion

This study suggested that indoor feeding with composite forage diet can improve the muscle color, tenderness and WHC of Black Tibetan sheep by regulating AA, lipid and carbohydrate metabolism in muscle tissues. In addition, indoor feeding regimes also had a positive effect on the composition of FAs and AAs in the LL. Further analyses showed that indoor feeding regimes even affected the levels of important metabolites (maltotriose, pyruvate, L-ascorbic acid, chenodeoxycholate, D-glucose 6-phosphate, glutathione, etc.), FAs, AAs and related metabolic pathways involved in regulating meat quality in Black Tibetan sheep’s LL. This was achieved through changes in the abundance of rumen bacteria (increased abundance of *Christensenellaceae R-7 group*, *[Eubacterium] coprostanoligenes group*, *Methanobrevibacter*, *Ruminococcus 2* and *Quinella*, decreased abundance of *Lactobacillus*, *Prevotella 1* and *Rikenellaceae RC9 gut group*, and the tendency for decreased abundance of *Selenomonas 1* and *Succinivibrio*). Altogether, the results suggested that indoor feeding regimes can improve the overall quality of Black Tibetan sheep’s meat.

## Data availability statement

The datasets presented in this study can be found in online repositories. The names of the repository/repositories and accession number(s) can be found at: NCBI SRA (accession: PRJNA895394). Access Links: http://www.ncbi.nlm.nih.gov/bioproject/895394.

## Ethics statement

This study was approved by the Animal Ethics Committee of Qinghai University (QUA-2020-0709) and was carried out at the Black Tibetan Sheep Breeding Center in Guinan County of Qinghai Province, China. Written informed consent was obtained from the owners for the participation of their animals in this study.

## Author contributions

XZ and LH: conceptualization, data curation, formal analysis, investigation, methodology, software, validation, writing-original draft, and writing-review and editing. SH and LG: data curation, formal analysis, visualization, software, validation, writing-review and editing, and funding acquisition. BY, ZW, YM, SR, RM, ZA, and SI: validation, visualization, writing-review and editing, and methodology. All authors have read and agreed to the published version of the manuscript.

## Funding

The current work was funded by Evaluation and Analysis of Nutritional Value of Black Tibetan Sheep and Research on Development of Series Products Funds of Qinghai Province (2020-GN-119) and Construction of Standardized Production System for Improving quality and efficiency of Tibetan sheep industry (2022-NK-169).

## Conflict of interest

The authors declare that the research was conducted in the absence of any commercial or financial relationships that could be construed as a potential conflict of interest.

## Publisher’s note

All claims expressed in this article are solely those of the authors and do not necessarily represent those of their affiliated organizations, or those of the publisher, the editors and the reviewers. Any product that may be evaluated in this article, or claim that may be made by its manufacturer, is not guaranteed or endorsed by the publisher.

## Footnotes

^1^ www.genome.jp/kegg
