# Supplementary material for: Metabolome and microbiome analysis revealed the effect mechanism of different feeding modes on the meat quality of Black Tibetan sheep
Source: Front Microbiol. 2023 Jan 6;13:1076675. doi: 10.3389/fmicb.2022.1076675 (PMC9854131; doi:10.3389/fmicb.2022.1076675)
Supplement: Supplementary file 1 [file Data_Sheet_1.docx]

Supplementary Material

# Supplementary Figures and Tables

## Supplementary Figures


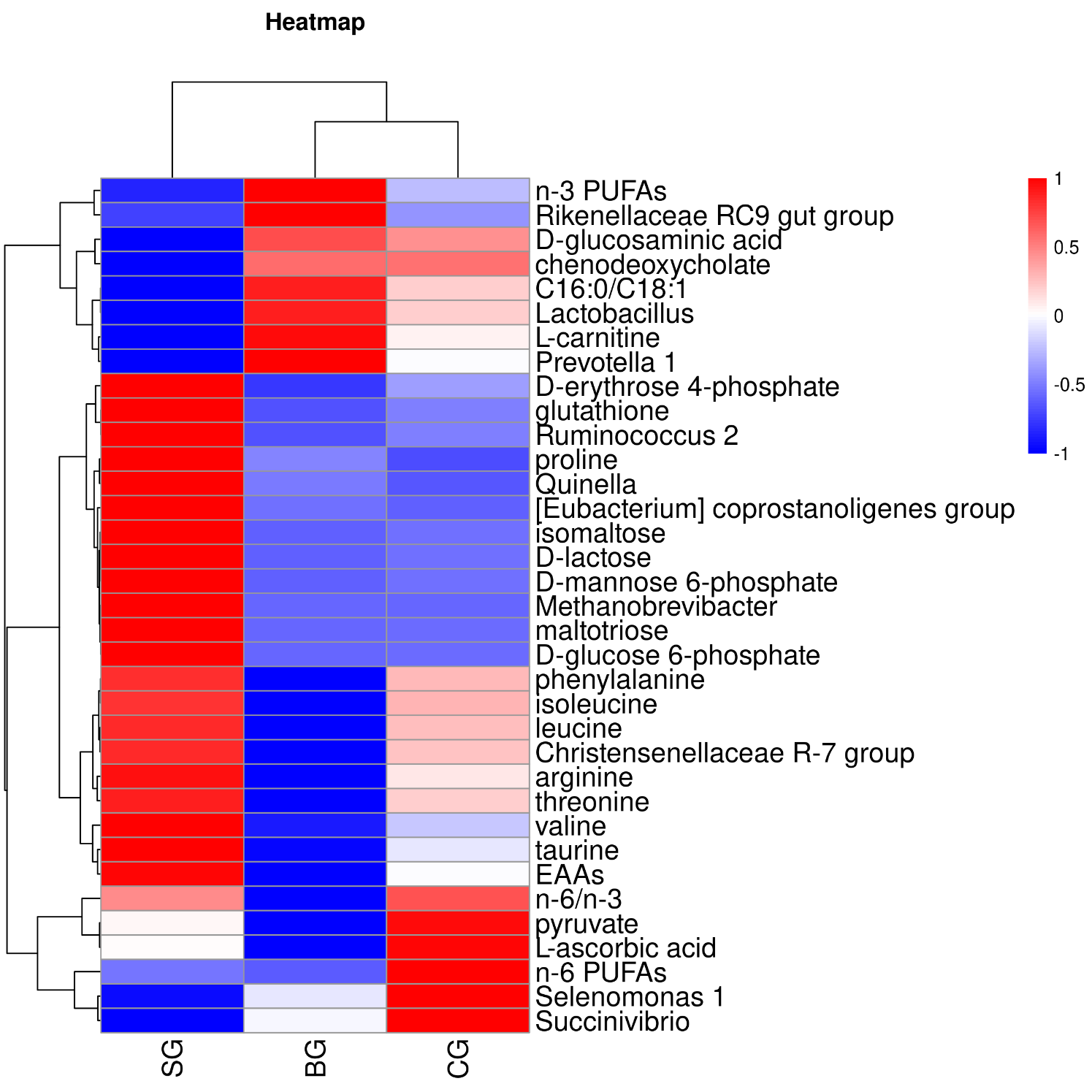


**Supplementary Figure 1.** Heatmap for the expression levels of important AAs, FAs, metabolites and bacteria in SG, BG and CG.

## Supplementary Tables

**Supplementary Table 1.** Evaluation parameters of OPLS-DA models in the positive ion mode.

|  | R^2^X (cum) | R^2^Y (cum) | Q^2^ (cum) |
| --- | --- | --- | --- |
| SG vs BG | 0.655 | 0.989 | 0.898 |
| SG vs CG | 0.424 | 0.994 | 0.906 |
| BG vs CG | 0.746 | 0.998 | 0.709 |

**Supplementary Table 2.** Effect of different feeding redimes on alpha diversity of rumen bacteria of Black Tibetan sheep.

| Items | Groups | | | SEM | | *P*-value | |  |
| --- | --- | --- | --- | --- | --- | --- | --- | --- |
|  | SG | BG | CG | |  | |  | |
| shannon | 6.49^b^ | 7.53^a^ | 6.03^b^ | | 0.26 | | < 0.01 | |
| simpson | 0.95^b^ | 0.98^a^ | 0.92^c^ | | 0.01 | | < 0.01 | |
| chao1 | 1513.78^a^ | 1583.17^a^ | 1321.34^b^ | | 57.93 | | < 0.01 | |
| ace | 1565.03^a^ | 1658.75^a^ | 1381.06^b^ | | 60.61 | | < 0.01 | |

^a, b, c^ different superscripts within a row indicate significantly different values for *P*-value < 0.05.

**Supplementary Table 3.** DMs in the key metabolic pathways between the three comparisons (SG vs BG; SG vs CG; BG vs CG) in the *longissimus lumborum* of Black Tibetan sheep.

| metabolic pathways | metabolites |
| --- | --- |
| SG vs BG | |
| upregulation in the SG group | upregulation/downregulation in the SG group |
| Pentose phosphate pathway | glyceric acid, pyruvate, D-erythrose 4-phosphate / D-glucosaminic acid |
| Phenylalanine metabolism | phenaceturic acid, 3-hydroxyphenylacetic acid, pyruvate / hippuric acid |
| Arginine and proline metabolism | pyruvate, urea, L-hydroxyarginine, creatinine / |
| Taurine and hypotaurine metabolism | pyruvate, taurine / |
| Valine, leucine, and isoleucine biosynthesis | pyruvate, L-isoleucine / |
| Carbohydrate digestion and absorption | maltotriose, D-lactose / |
| D-Alanine metabolism | Pyruvate / |
| Ascorbate and aldarate metabolism | pyruvate, L-ascorbic acid / |
| Glycine, serine, and threonine metabolism | glyceric acid, pyruvate / |
| Phosphotransferase system (PTS) | D-lactose, pyruvate, L-ascorbic acid / D-glucosaminic acid |
| HIF-1 signaling pathway | pyruvate, L-ascorbic acid / |
| SG vs CG | |
| upregulation in the SG group | upregulation/downregulation in the SG group |
| Carbohydrate digestion and absorption | maltotriose, D-lactose, D-glucose 6-phosphate / |
| Arginine and proline metabolism | L-hydroxyarginine, urea, creatinine / |
| Starch and sucrose metabolism | isomaltose, D-glucose 6-phosphate / |
| Phosphotransferase system (PTS) | D-mannose 6-phosphate, D-lactose, D-glucose 6-phosphate / D-glucosaminic acid |
| Thyroid hormone synthesis | glutathione, D-glucose 6-phosphate / |
| downregulation in the SG group | upregulation/downregulation in the SG group |
| Bile secretion | glutathione / chenodeoxycholate, choline, L-carnitine |
| BG vs CG | |
| upregulation in the CG group | upregulation/downregulation in the CG group |
| Taurine and hypotaurine metabolism | taurocholate, pyruvate, taurine / |
| Valine, leucine, and isoleucine biosynthesis | pyruvate, L-isoleucine / |
| Pentose phosphate pathway | pyruvate, glyceric acid / |
| Valine, leucine, and isoleucine degradation | methylmalonic acid, L-isoleucine / |
| D-Alanine metabolism | pyruvate / |
| Ascorbate and aldarate metabolism | L-ascorbic acid, pyruvate / |
| Sphingolipid metabolism | ceramide (d18:1/18:1(9Z)), sphingomyelin (d18:1/18:0) / |
| Primary bile acid biosynthesis | taurocholate, glycocholic acid, taurine / |
| Secondary bile acid biosynthesis | taurocholate, glycocholic acid / |
| Cholesterol metabolism | taurocholate, glycocholic acid / |
| HIF-1 signaling pathway | L-ascorbic acid, pyruvate / |
| downregulation in the CG group | upregulation/downregulation in the CG group |
| beta-Alanine metabolism | / pantothenate, aspartic acid, anserine |
| Histidine metabolism | / aspartic acid, anserine |

**Supplementary Table 4.** Effect of different feeding modes on the AA composition in the *longissimus lumborum* of Black Tibetan sheep (μg/g tissue).

| Items | Groups | | | SEM | *P*-value |
| --- | --- | --- | --- | --- | --- |
|  | SG | BG | CG |  |  |
| glutamate | 47.35 | 36.38 | 79.45 | 31.88 | 0.43 |
| glycine | 323.20 | 362.28 | 280.03 | 53.15 | 0.37 |
| lysine | 44.70 | 44.18 | 35.36 | 7.16 | 0.40 |
| aspartate | 8.22 | 71.68 | 17.84 | 26.31 | 0.10 |
| arginine | 108.46 | 66.26 | 90.77 | 14.61 | 0.07 |
| serine | 55.42 | 49.75 | 41.19 | 6.28 | 0.15 |
| methionine | 5.87^a^ | 4.07^b^ | 5.71^a^ | 0.35 | < 0.01 |
| phenylalanine | 44.25^a^ | 26.90^b^ | 39.47^a^ | 3.22 | < 0.01 |
| tyrosine | 47.02^a^ | 27.64^b^ | 38.63^ab^ | 5.86 | 0.04 |
| leucine | 74.33^a^ | 47.42^b^ | 66.43^a^ | 5.74 | < 0.01 |
| isoleucine | 23.84^a^ | 17.04^b^ | 22.02^a^ | 1.32 | < 0.01 |
| histidine | 68.88 | 97.71 | 95.37 | 23.46 | 0.44 |
| proline | 77.08^a^ | 40.81^b^ | 35.98^b^ | 10.40 | 0.01 |
| valine | 54.07^a^ | 37.86^b^ | 43.56^b^ | 4.26 | 0.02 |
| threonine | 57.63 | 22.92 | 45.43 | 12.53 | 0.08 |
| alanine | 542.20 | 276.66 | 496.99 | 120.95 | 0.14 |
| asparagine | 43.93^a^ | 20.48^b^ | 34.95^ab^ | 6.26 | 0.03 |
| creatine | 1051.36 | 1138.96 | 1138.05 | 45.21 | 0.16 |
| citrulline | 43.14 | 60.94 | 28.53 | 14.95 | 0.18 |
| glutamine | 1212.64 | 686.30 | 1705.48 | 514.41 | 0.22 |
| cysteine | 0.30 | 1.66 | 1.66 | 0.85 | 0.26 |
| creatinine | 13.26 | 12.00 | 12.20 | 0.58 | 0.15 |
| tryptophan | 39.51^b^ | 54.88^a^ | 42.05^b^ | 4.91 | 0.04 |
| hydroxyproline | 36.43^a^ | 10.80^b^ | 36.59^a^ | 7.94 | 0.03 |
| ornithine | 39.99 | 8.54 | 13.27 | 13.51 | 0.12 |
| taurine | 1014.74^a^ | 365.16^b^ | 678.34^ab^ | 188.75 | 0.04 |
| choline | 22.14 | 190.63 | 50.89 | 64.24 | 0.08 |
| aminoadipic acid | 26.24^a^ | 9.19^b^ | 16.09^ab^ | 5.31 | 0.05 |
| EAAs ^1^ | 344.21 | 255.26 | 300.03 | 33.19 | 0.09 |
| NEAAs | 4781.98 | 3533.80 | 4892.30 | 798.59 | 0.25 |
| TAAs | 5126.19 | 3789.06 | 5192.33 | 830.41 | 0.24 |

^a, b^ different superscripts within a row indicate significantly different values for *P*-value < 0.05. EAAs: essential amino acids, NEAAs: non-essential amino acids, TAAs: total amino acids.

^1^ EAAs = (leucine + methionine + valine + isoleucine + threonine + phenylalanine + lysine + tryptophan)

**Supplementary Table 5.** Effect of different feeding modes on the FA composition in the *longissimus lumborum* of Black Tibetan sheep (μg/g tissue).

| Items | Groups | | | SEM | *P*-value |
| --- | --- | --- | --- | --- | --- |
|  | SG | BG | CG |  |  |
| C6:0 | 0.04 | 0.04 | 0.06 | 0.02 | 0.57 |
| C10:0 | 1.01 | 1.65 | 1.16 | 0.75 | 0.70 |
| C11:0 | 0.02 | 0.09 | 0.08 | 0.06 | 0.45 |
| C12:0 | 3.87 | 12.75 | 12.60 | 7.85 | 0.48 |
| C13:0 | 0.23 | 0.77 | 1.03 | 0.52 | 0.35 |
| C14:0 | 130.07 | 211.77 | 185.81 | 93.63 | 0.69 |
| C14:1N5 | 4.38 | 5.61 | 3.52 | 2.26 | 0.67 |
| C15:0 | 12.22 | 17.28 | 32.52 | 11.57 | 0.27 |
| C15:1N5 | 3.49 | 4.68 | 7.74 | 2.54 | 0.30 |
| C16:0 | 1560.05 | 1441.80 | 1635.60 | 607.10 | 0.95 |
| C16:1N7 | 130.02 | 75.24 | 101.33 | 55.32 | 0.64 |
| C17:0 | 49.03 | 45.84 | 88.80 | 29.66 | 0.34 |
| C17:1N7 | 40.22 | 29.20 | 49.95 | 16.11 | 0.48 |
| C18:0 | 1237.42 | 1000.61 | 1456.91 | 526.62 | 0.70 |
| C18:1N9 | 2630.31 | 1790.71 | 2098.61 | 858.51 | 0.64 |
| C18:2N6 | 520.10 | 515.18 | 706.96 | 76.09 | 0.08 |
| C18:3N6 | 6.20^a^ | 3.19^b^ | 7.01^a^ | 1.09 | 0.03 |
| C18:3N3 | 26.54 | 30.90 | 52.72 | 9.59 | 0.07 |
| C20:0 | 8.67 | 7.14 | 10.81 | 3.68 | 0.63 |
| C20:1N9 | 22.48^a^ | 10.94^b^ | 13.22^b^ | 2.09 | < 0.01 |
| C21:0 | 3.61 | 3.40 | 3.44 | 0.37 | 0.84 |
| C20:3N6 | 18.68 | 28.17 | 25.19 | 3.65 | 0.10 |
| C20:4N6 | 2.81 | 2.07 | 2.56 | 0.24 | 0.06 |
| C20:3N3 | 187.83 | 310.29 | 190.08 | 47.26 | 0.07 |
| C22:0 | 1.51 | 1.53 | 2.24 | 0.47 | 0.28 |
| C20:5N3 | 14.40^b^ | 44.02^a^ | 21.83^ab^ | 9.45 | 0.05 |
| C22:1N9 | 8.26 | 4.63 | 5.42 | 1.93 | 0.22 |
| C22:2N6 | 0.38 | 0.30 | 0.30 | 0.15 | 0.81 |
| C23:0 | 0.88 | 0.72 | 1.17 | 0.17 | 0.10 |
| C22:4N6 | 27.53 | 18.07 | 26.48 | 3.91 | 0.10 |
| C22:5N6 | 8.02 | 6.27 | 6.71 | 1.03 | 0.29 |
| C24:0 | 1.60 | 1.73 | 2.31 | 0.36 | 0.19 |
| C22:5N3 | 39.09^b^ | 88.59^a^ | 69.11^ab^ | 12.97 | 0.02 |
| C22:6N3 | 6.77^b^ | 22.67^a^ | 8.90^b^ | 4.48 | 0.02 |
| TFAs | 6707.74 | 5737.86 | 6832.17 | 2164.09 | 0.86 |
| SFAs | 3010.23 | 2747.13 | 3434.54 | 1250.24 | 0.86 |
| MUFAs | 2839.16 | 1921.01 | 2279.78 | 931.81 | 0.63 |
| PUFAs | 858.35 | 1069.72 | 1117.85 | 137.12 | 0.21 |
| n-3 PUFAs | 274.63 | 496.47 | 342.64 | 71.72 | 0.05 |
| n-6 PUFAs | 583.73 | 573.25 | 775.21 | 78.04 | 0.07 |
| PUFAs/SFAs | 0.34 | 0.45 | 0.39 | 0.16 | 0.80 |
| n-6 PUFAs/n-3 PUFAs | 2.12^a^ | 1.19^b^ | 2.26^a^ | 0.17 | < 0.01 |
| C16:0/C18:1 | 0.58^b^ | 0.87^a^ | 0.77^ab^ | 0.09 | 0.05 |

^a, b^ different superscripts within a row indicate significantly different values for *P*-value < 0.05. TFAs: total fatty acids, SFAs: saturated fatty acids, MUFAs: monounsaturated fatty acids, PUFAs: polyunsaturated fatty acids, n-3 PUFAs: omega-3 polyunsaturated fatty acids, n-6 PUFAs: omega-6 polyunsaturated fatty acids.
